# UPLC‐ESI‐QTOF‐MS/MS Profiling, Antioxidant, and Cytotoxicity Potentials of *Marrubium vulgare* L. Extracts: Experimental Analysis and Computational Validation

**DOI:** 10.1002/cbdv.202500400

**Published:** 2025-06-14

**Authors:** Ines El Mannoubi, Nuha M. Alghamdi, Seham H. Bashir, Suada Alsaied Mohamed, Hedia Chaabane, Ashraf N. Abdalla, Majdi Abid, Adel Kadri, Mozaniel Santana de Oliveira

**Affiliations:** ^1^ Chemistry Department, Faculty of Science Al‐Baha University Al‐Baha Kingdom of Saudi Arabia; ^2^ Research Unit Advanced Materials, Applied Mechanics, Innovative Processes, and Environment, Higher Institute of Applied Sciences and Technology of Gabes University of Gabes Tunisia; ^3^ Department of Chemistry and Industrial Chemistry, College of Applied and Industrial Sciences University of Bahri Sudan; ^4^ Laboratory of Natural Substances National Institute of Research and Physico‐Chemical Analysis Ariana Tunisia; ^5^ Department of Pharmacology and Toxicology, College of Pharmacy Umm Al‐Qura University Makkah Kingdom of Saudi Arabia; ^6^ Department of Pharmacology and Toxicology Medicinal and Aromatic Plants Research Institute, National Center for Research Khartoum Sudan; ^7^ Department of Chemistry, College of Science Jouf University Sakaka Kingdom of Saudi Arabia; ^8^ Faculty of Science of Sfax, Department of Chemistry University of Sfax Sfax Tunisia; ^9^ Postgraduate Program in Pharmaceutical Sciences (PPGCF) Institute of Health Sciences, Federal University of Pará Belém Brazil

**Keywords:** antioxidant, cytotoxicity, in silico study, *Marrubium vulgare* L., UPLC‐ESI‐QTOF‐MS/MS

## Abstract

Plant extracts are emerging as valuable options for food additives and therapeutic treatments. This study evaluated the phytochemical profile, antioxidant activity, and cytotoxicity of aerial parts of *Marrubium vulgare* L. crude extract (MVCE) and its subfractions. The MVCE (80% ethanol) contained steroids, phenolic compounds, flavonoids, terpenes, and cardiac glycosides, with total phenolic content (TPC) and total flavonoid content (TFC) of 14.96 ± 0.12 mg GAE/g DW and 12.27 ± 0.63 mg RE/g DW, respectively. All MV extracts exhibited potent antioxidant activity against DPPH^•^ (0.106–1.864 mg/mL) and ABTS^+•^ (0.298–17.084 mg/mL). The MV residual aqueous fraction (MVRF) showed significant cytotoxicity against human cancer cell lines, including MCF7 (IC_50_ = 5.47 ± 1.32 µg/mL), HT29 (IC_50_ = 17.48 ± 1.47 µg/mL), and SW480 (IC_50_ = 7.51 ± 0.36 µg/mL). Ultra‐performance liquid chromatography‐mass spectrometry identified 26 bioactive compounds, with malic acid, caffeic acid, chlorogenic acid, kaempferol‐3‐glucuronide, and l‐tryptophan as the major ones. Molecular docking revealed strong binding affinities of the above compounds to breast (PDB ID: 6CHZ) and colorectal cancer (PDB ID: 1HVY) proteins. Pharmacokinetic and toxicological studies confirmed their safety and efficacy, supporting MVRF as a potential therapeutic agent. These findings highlight MV as a promising candidate for future anticancer research.

## Graphical abstract

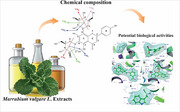

## 1 Introduction

Cancer is a significant public health concern at the global level, identified as a noncommunicable and multifactorial burden [1, 2]. As indicated by the Global Cancer Observatory (GCO) database, cancer represents one of the primary causes of mortality in Gulf Cooperation Council (GCC) countries [3]. In 2022, Saudi Arabia had the highest global accounting number of new cases (28 113) and the highest number of deaths (13 399) due to cancer. The estimated 5‐year prevalent cases were 46 288 in males and 48 663 in females [4]. Breast cancer (BC) and colorectal cancer (CRC) are the most commonly prevalent malignancies and the leading causes of cancer death for both sexes in Saudi Arabia, accounting for 3777 (13.4%)/1046 and 3750 (13.3%)/1883 new cases/deaths, respectively, of all cancer incidence [5]. An excessive and aberrant formation of reactive oxygen species (ROS) above the cellular tolerability threshold, followed by a weakening of the immune defense system, can interfere with cancer pathology [6, 7, 8].

The elimination of these cells through the enhancement of scavenging abilities represents a promising strategy for cancer therapies [6, 7, 8]. Conventional cancer therapies include surgical procedures, radiation, hormonal and targeted therapies, and chemotherapy, which often result in significant adverse side effects [9, 10]. Doxorubicin (DOX) is an effective chemotherapeutic drug that is widely recognized as one of the most popular drugs used to treat metastatic human BC [11, 12]. In contrast, contemporary traditional pharmacopoeias derived from plant‐derived biomolecules have emerged as promising strategies for safer and more effective therapeutic purposes, with the objective of preventing or curing cancer through the discovery of potential anticancer drugs [13, 14, 15, 16].

*Marrubium vulgare* L. (MV), a member of the Lamiaceae family, is an annual indigenous plant commonly known as the white horehound. It has a naturalized distribution in North Africa, Europe, and Central Asia [17]. The plant has traditionally been reported to have a range of therapeutic effects, including the treatment of inflammation, pulmonary infections, rheumatoid arthritis, night blindness, and stomach disorders [18, 19, 20]. Phytochemical analysis revealed the abundance of diterpenes (labdanes), particularly marrubenol and marrubiin [18, 19, 20, 21, 22]. Extracts from this plant provide a valuable source of bioactive molecules that can be utilized in the development of novel drugs.

The *Marrubium* genus plants have been demonstrated to possess high phenolic contents, comprising the majority of classes, including phenylpropanoid and phenolic acids, esters, flavonoids, flavone derivatives (flavone aglycones and flavone glycosides), and tannins [23, 24]. In addition, the presence of nitrogen‐containing compounds, polysaccharides, phytosterols, amino acids, organic acids, alkaloids, and minerals, in particular potassium salts in MV has been documented [25, 26, 27]. The high efficiency of these phytochemicals for various purposes and their known beneficial pharmacological attributes have led to this plant being designated a “plant drug” for use in cancer therapy and general health. Biological investigations have confirmed the analgesic, antioxidant, diuretic, hypoglycemic, hypolipidemic, antihypertensive, antidiabetic, diaphoretic, antinociceptive, antispasmodic, gastroprotective, anti‐inflammatory, antimicrobial, vasodilating, and antiproliferative effects of this plant [22, 28, 29]. As an herbal beverage, it was frequently incorporated into teas, syrups, and other preparations. It was also used as a bitter and choleretic, as well as for ovoid dyspeptic complaints and in food applications [30].

The present study was designed to elucidate the phytoconstituents and metabolite profiling of MV extract(s) through the use of an untargeted metabolomic approach, employing ultrahigh‐performance liquid chromatography with quadrupole time‐of‐flight and mass spectrometry (UHPLC‐QTOF‐MS/MS). The antioxidant and cytotoxic effects of the extracts were evaluated, while molecular docking and pharmacokinetic analysis of the selected major phytocompounds from MV residual aqueous fraction (MVRF), which underline the potent anticancer property, were performed.

## 2 Results and Discussion

### 2.1 Phytochemical Screening, Total Phenolic Content, and Total Flavonoid Content

The qualitative phytochemical screening of the MV crude extract (MVCE) revealed the presence of steroids, phenolic compounds, flavonoids, terpenes, and cardiac glycosides, but the absence of alkaloids and saponins (results not shown). The total phenolic content (TPC) and total flavonoid content (TFC) in the MVCE were found to be 14.96 ± 0.12 mg GAE/g dry weight (DW) and 12.27 ± 0.63 mg RE/g DW, respectively. Some recent studies have focused on the TPC and TFC of this species. Michalak et al. demonstrated a TPC of 55.72 mg GAE/mL and a TFC of 11.01 mg catechin equivalent/mL in a 50/50 ethanol/water (v/v) extract from aerial parts of MV collected from Poland using ultrasonication [31]. Emam et al. determined TPC by spectrophotometry and obtained 240.46 ± 12.19 mg of GAE/g DW of phenolic content in a 70% ethanol extract from macerated aboveground parts originating from Egypt for 3 weeks [32]. The hot infusion of aerial parts of MV from Tunisia exhibited a richness in phenolic content (73.619 mg GAE/g extract) [33]. Gavarić et al. reported that the TPC and TFC in MV extracts are influenced by the extraction method in the following descending order: microwave extraction > ultrasound‐assisted extraction > conventional solid‐liquid extraction. TPC ranged from 63.77 to 117.58 mg GAE/g DW, while TFC ranged from 42.51 to 65.80 mg catechin equivalent/g DW [34]. On the other hand, Khouchlaa et al. reported that the TPC and TFC of the methanolic extract of MV aerial parts from Morrocco did not exceed 0.0430 mg GAE/g DW and 1.644 mg quercetin/g DW, respectively, during three development stages [35]. Meanwhile, the methanolic extract of MV leaves collected from the Al‐Taif Governorate (KSA) showed a TPC of 36.8 mg GAE/g DW [36].

Overall, the above results reveal that the content of secondary metabolites is influenced by genetics, geographical and environmental factors, regional climatic conditions, the type of solid/liquid extraction (common/green), the kind of extract (organic/aqueous), and the polarity index of the solvent [34, 37–39].

### 2.2 Antioxidant Activity

The MVCE and its subfractions exhibited concentration‐dependent DPPH^•^ and ABTS**^+•^** radical scavenging activities expressed as IC_50_ values (mg/mL) summarized in Table 1. Our findings demonstrate that all extracts significantly reduce DPPH^•^ radical to varying degrees (*p* < 0.05). MVPF (1.864 ± 0.069 mg/mL) is the least effective, followed by MVCF and MVRF, with no observable significant difference, followed by MVCE, significantly less effective than MVEF. The latter fraction showed the highest radical quenching potential among all extracts, but it was comparable to the positive control (Trolox, 0.012 ± 0.000 mg/mL) with values of 0.106 ± 0.008 mg/mL as the difference in IC_50_ values was not significant (*p* = 0.161). The effects of MVCE and subfractions on the ABTS^+•^ radical were also studied. All extracts were able to quench the ABTS^+•^ cationic radical except for MVPF (17.084 ± 1.101 mg/mL). The effectiveness of the crude extract and corresponding subfractions as ABTS^+•^ quenchers were ranked in the following increasing order MVCF (3.375 ± 0.120 mg/mL) < MVRF (1.727 ± 0.018 mg/mL), MVCE (1.473 ± 0.075) (*p* = 0.980) ≤ MVEF (0.298 ± 0.027), Trolox (0.055 ± 0.000 mg/mL) (*p* = 0.984).

**TABLE 1:** DPPH (IC_50_, mg/mL) and ABTS**^+•^** (IC_50_, mg/mL) of *Marrubium vulgare* L. aerial parts crude extract and subfractions.

Sample	Solvent	DPPH^•^	ABTS^+•^
MVCE	EtOH (80%)	0.349 ± 0.072^c^	1.473 ± 0.075^cd^
MVPF	Petroleum ether	1.864 ± 0.069^a^	17.084 ± 1.101^a^
MVCF	Chloroform	0.656 ± 0.033^b^	3.375 ± 0.120^b^
MVEF	Ethyl acetate	0.106 ± 0.008^d^	0.298 ± 0.027^de^
MVRF	Residual aqueous fraction	0.544 ± 0.017^b^	1.727 ± 0.018^c^
Trolox	—	0.012 ± 0.000^d^	0.055 ± 0.000^e^

*Note*: Values are mean of three replicates ± standard deviation (*n* = 3). Different letters in the same column indicate a significant difference according to the Tukey test at *p* < 0.05.

Overall, we can conclude that only MVCE, MVEF, and MVRF worked excellently against both DPPH^•^ and ABTS^+•^ to reduce stable radicals among all studied extracts. The descending order of radical scavenging potential was as follows: MVEF > MVCE > MVRF, but they were still less effective than the positive control. This can be explained by the abundance of phytochemicals in these extracts serving as potential hydrogen donors that induce the reduction of free radicals [40]. DPPH^•^ and ABTS**^+•^** assays are both hydrogen atom transfer processes working on an identical mechanism [41]. Therefore, it is evident that they revealed consistent results.

Recently, Duc et al. attributed the effectiveness of phenolic compounds as oxidation inhibitors to their chemical structure [42]. The presence and location of hydroxyl groups on the aromatic ring are important, as they contribute to the low bond dissociation energy and strength in both phenolic acids and flavonoids. Furthermore, the effectiveness of flavonoids in neutralizing free radicals is related to the presence of a double bond between C‐2 and C‐3 in conjugation with a carbonyl group at C‐4 and the absence of sugar units at C‐3. Previous studies reported the capacity of MV to quench DPPH^•^ and ABTS**^+•^** radicals due to the presence of several potent antioxidants such as phenylethanoid glycosides, tannins, flavonoids, and phenolic acids [43].

#### 2.2.1 Correlation Between Phenolic Compounds and Antioxidant Activity

In this study, a Pearson correlation analysis was conducted to explore the relationships between the TPC and TFC and antioxidant activity (DPPH^•^ and ABTS^+•^) of the MVCE. The objective of this analysis was to ascertain the manner in which alterations in phenolic and flavonoid concentrations are associated with the plant's antioxidant capabilities, thereby offering insight into the contribution of these compounds to the plant's overall salutary effects. As illustrated in Table 2, there is a robust correlation between TPC and TFC (*r* = 0.887). The correlation coefficients between DPPH^•^ and (TPC and TFC) were *r* = −0.852 (*p* > 0.05) and −0.998 (*p* < 0.05), respectively, indicating a significant negative correlation between phenolic acids and flavonoids and DPPH^•^ inhibition. Furthermore, a moderate positive correlation was identified between ABTS**^+•^** and TPC (*r* = 0.439, *p* > 0.05), while a strong positive correlation was observed between ABTS**^+•^** and TFC (*r* = 0.805, *p* > 0.05).

**TABLE 2:** Pearson's correlation test (*r* values) between TPC, TFC, and antioxidant capacity (DPPH^•^ and ABTS^+•^ assays) for MVCE.

	TPC	TFC	DPPH^•^	ABTS^+•^
TPC	1	0.887	−0.852	0.439
TFC		1	−0.998^*^	0.805
DPPH**^•^**			1	−0.845
ABTS**^+•^**				1

* Significant correlation at *p* ≤ 0.05.

This contrasting antioxidant behavior may be explained by factors such as radical stability, reaction mechanism, and the structural properties of polyphenols, including basic structure, hydroxyl group number and position, and Bors criteria [44, 45]. It is also important to consider other factors, such as pH, solvent, carbohydrate, and protein content, as these can influence the reported activity of natural extracts [46]. In conclusion, TPC and TFC exhibited a strong correlation with the DPPH^•^ assay compared to the ABTS^+•^ assay. This underscores the significant role of phenolics and flavonoids in antioxidant capacity, especially in terms of DPPH^•^ radical scavenging activity.

### 2.3 Cytotoxicity on Normal and Cancerous Cells

The cytotoxicity of the crude extract of MV and its subfractions was evaluated after 72 h of incubation on three cancerous cell lines (MCF7, HT29, and SW480) and one non‐cancerous MRC5 cell line. The IC_50_ values (µg/mL) were determined through the use of the MTT assay. All extracts exhibited dose‐dependent cytotoxic activity against the three tested cancerous cell lines. As shown in Table 3, the crude extract and its five subfractions exhibited notable cytotoxicity against the aforementioned cell lines, with *p* values less than 0.05. The results demonstrated notable discrepancies among the cell lines and extracts, with some exhibiting comparatively diminished activity relative to DOX.

**TABLE 3:** Cytotoxic activity of *Marrubium vulgare* crude extract and subfractions against three cell lines and normal fibroblast (MTT 72 h, IC_50_ “µg/mL” ± SD, *n* = 3).

Sample	Solvent	MCF7	HT29	SW480	MRC5
MVCE	EtOH (80%)	13.60 ± 0.90^d^	44.78 ± 2.70^a^	4.92 ± 0.77^d^	20.84 ± 0.34^c^
MVPF	Petroleum ether	18.83 ± 0.46^c^	15.85 ± 0.46^c^	25.05 ± 1.93^b^	21.52 ± 0.38^c^
MVCF	Chloroform	22.06 ± 0.73^b^	28.29 ± 2.07^b^	35.35 ± 1.15^a^	22.38 ± 0.37^c^
MVEF	Ethyl acetate	24.68 ± 1.40^a^	26.32 ± 2.31^b^	14.81 ± 1.92^c^	45.27 ± 1.90^a^
MVRF	Residual aqueous fraction	5.47 ± 1.32 ^e^	17.48 ± 1.47^c^	7.51 ± 0.36^d^	35.77 ± 3.45^b^
Doxorubicin	—	0.04 ± 0.00^f^	0.05 ± 0.01^d^	0.06 ± 0.01^e^	2.48 ± 0.02^d^

*Note*: Average cytotoxicity (IC_50_) of each extract against the three cancer cells. Values are mean of three replicates ± standard deviation (*n* = 3). Different letters in the same column indicate a significant difference according to the Tukey test (*p* < 0.05).

The IC_50_ values ranged from 5.47 ± 1.32 to 24.68 ± 1.40 µg/mL against MCF7. The order of the cytotoxic effects of the extracts is as follows: MVEF < MVCF < MVPF < MVCE < MVRF. With regard to HT29, the IC_50_ values exhibited a range of 15.85 ± 0.46–44.78 ± 2.70 µg/mL. The results demonstrated that MVCE was less potent than MVCF and MVEF (p = 0.756), which in turn were less potent than MVPF and MVRF (*p* = 0.866). Against SW480, the IC_50_ values ranged from 7.51 ± 0.36 to 35.35 ± 1.15 µg/mL. The order of the cytotoxic effects of the extracts was as follows: The order of the cytotoxic effects of the extracts was as follows: MVCF < MVPF < MVEF < MVCE, MVRF (*p* = 0.191). In contrast, the crude extract and its subfractions demonstrated a moderate to weak impact on the healthy MRC5 cell line, with IC_50_ values ranging from 13.29 ± 0.92 to 45.27 ± 1.90 µg/mL. The observed effect was found to diminish in the following descending order: MVCE, MVPF, and MVCF (*p* = 0.849) exhibited greater potency than MVRF and MVEF. As can be observed, MRC5 demonstrates reduced sensitivity to MV extract action, which is an anticipated outcome given that these normal cells are untransformed and of healthy connective tissue cell nature [47]. In general, human breast and colorectal cell lines demonstrate greater sensitivity to polar fractions than less polar fractions. MVRF demonstrated the greatest efficacy against all cancerous cell lines. It can thus be concluded that the adopted fractionation process resulted in the production of fractions with greater activity than the crude extract. These results can be attributed to the disparities in phytochemical composition, contingent upon the fraction's polarity in relation to the solvent extraction type and polarity index.

### 2.4 UPLC‐ESI‐QTOF‐MS/MS Characterization of the Residual Aqueous Fraction of MV

The phytochemical constituents of the MVRF were tentatively identified by means of UHPLC‐QTOF‐MS/MS. In addition, the HMDB (http://www.hmdb.ca/) and Mass Bank (https://massbank.eu/MassBank/) databases were consulted for the purpose of identification, utilizing the precise masses of the molecules in question. In total, 26 peaks were identified, with 11 in the ESI negative mode, 12 in the ESI positive mode, and 3 compounds identified in both modes. The results are presented in Table 4, and the total ion chromatograms (TICs) for the ESI^−^ and ESI^+^ modes are displayed in supplementary material Figure S1a,b, respectively. The identified secondary metabolites belong to various natural product classes, including three organic acids, two phenolic acids, six flavonoid glycosides, and other compounds, as detailed in Table 4. The MS/MS spectra and structures of some identified compounds are presented in Figures S2 and S3, respectively.

**TABLE 4:** UPLC‐ESI‐QTOF‐MS/MS metabolic analysis of MVRF.

No.	Compound	RT (min)	Experimental (*m*/*z*)	Area	Error ppm	Ionization (ESI^+^/ESI^−^)	Theoretical (*m*/*z*)	Formula	MS^2^ ion products	References
**Organic acids**										
1	Succinic acid	1.008	117.0195	23 623.3	−1.2	[M−H]^−^	117.01933	C_4_H_6_O_4_	55.02012 73.03007 99.00807	[48, 49]
2	Malic acid	1.046	133.0128	67 580.6	−1.8	[M−H]^−^	133.0142	C_4_H_6_O_5_	71.0134 89.0231 115.0024	[50, 51, 52]
3	Citraconic acid	1.474	128.96	70 409.3	−0.4	[M−H]^−^	129.01933	C_5_H_6_O_4_	84.98897	MassBank
**Phenolic acids**										
4	Caffeic acid	1.087	179.0569	33 793.7	−0.7	[M−H]^−^	179.03499	C_9_H_8_O_4_	71.01284 75.00822 89.0319 99.00691 143.03537 161.04589	[49, 53–57]
5	Chlorogenic acid	6.087	355.1726	17 164.8	1.2	[M+H]^+^	355.10236	C_16_H_18_O_9_	163.0387	[58, 59, 60, 61]
**Flavonoids**										
**Flavonols**										
6	Quercitrin	1.309	447.1172	6709.2	0.5	[M−H]^−^	447.09329	C_21_H_20_O_11_	149.03605 151.05052 152.01283 299.08168	[62]
7	Kaempferol‐3‐glucuronide	6.251	461.074	71 500.5	1.3	[M−H]^−^	461.07254	C_21_H_18_O_12_	175.03933 285.04057	[62, 63, 64, 65]
9.756		463.0881	103 489.3	0.5	[M+H]^+^	463.0871	C_21_H_18_O_12_	287.05566	[66]
8	Isorhamnetin‐3‐*O*‐rutinoside	7.484	623.2025	27 863.4	−0.4	[M−H]^−^	623.16174	C_28_H_32_O_16_	161.02477 315.10612 461.16721 623.19975	[65, 67]
**Flavones**										
9	Vicenin‐2	5.785	593.1548	10 424.5	0.8	[M−H]^−^	593.15118	C_27_H_30_O_15_	353.06696 383.07783 473.11029 593.1507	[68, 69, 70, 71, 72, 73, 74]
		6.117	595.1658	8988.8	−0.4	[M+H]^+^	595.16577	C_27_H_30_O_15_	295.05884 325.07021 337.06948 379.08152 457.11172 529.1370 559.14537 577.15467	[75, 76]
10	Baicalein‐7‐*O*‐glucuronide	6.732	445.0813	10 455.9	−0.7	[M−H]^−^	445.07764	C_21_H_18_O_11_	175.02541 269.04758	[65, 72, 77]
**Flavanones**										
11	Naringenin‐7‐*O*‐glucoside	8.845	435.1279	8836.5	0.7	[M+H]^+^	435.12857	C_21_H_22_O_10_	273.07332	[78]
**Amino acids**										
12	l‐Tryptophan	4.303	203.0833	17 746.0	0	[M−H]^−^	203.0826	C_11_H_12_N_2_O_2_	72.00852 74.02491 116.05056 142.06585 159.09128 186.0536	[77]
4.521		205.0973	17 706.8	−1.6	[M+H]**^+^**	205.09715	C_11_H_12_N_2_O_2_	118.0647 146.0593 188.0700	[79]
13	l‐Ornithine	1.135	133.061	16 270.88	−1	[M+H]**^+^**	133.09715	C_5_H_12_N_2_O_2_	74.0257	HMDB
14	l‐Proline	1.236	116.0704	14 577.7	−1.4	[M+H]**^+^**	116.0706	C_5_H_9_NO_2_	70.0652	[80]
15	Tyrosine	1.261	182.0581	7844.0	0.2	[M+H]**^+^**	182.08118	C_9_H_11_NO_3_	91.0597	[79]
16	Norvaline	1.308	118.0866	18 930.7	−2.9	[M+H]**^+^**	118.08626	C_5_H_11_NO_2_	55.0571 72.0847	HMDB
**Bile acids**										
17	Cholic acid	14.050	409.1622	33 542.04	−0.1	[M+H]^+^	409.29486	C_24_H_40_O_5_	—	HMDB
**Triterpene saponins**										
18	Glycyrrhizin	23.218	821.5222	36 026.2	0.8	[M−H]^−^	821.39648	C_42_H_62_O_16_	—	[81, 82]
**Other compounds**										
19	Anserine	11.195	239.0683	9384.6	0	[M−H]^−^	239.11496	C_10_H_16_N_4_O_3_	176.03577 207.04123	HMDB
20	5‐Aminoimidazole‐4‐Carboxamide‐1‐ribofuranosyl 5′‐monophosphate	16.633	337.2076	12 561.2	−0.4	[M−H]^−^	337.05548	C_9_H_15_N_4_O_8_P	96.96002	HMDB
21	1‐Myristoyl‐2‐hydroxy‐sn‐glycero‐3‐phosphate	17.143	381.2335	7071.0	−0.9	[M−H]^−^	381.20477	C_17_H_35_O_7_P	96.95987	[83] HMDB
22	Nicotanamide	1.646	122.9235	20 565.8	5.5	[M+H]**^+^**	123.05529	C_6_H_6_N_2_O	80.0477	[84]
23	*S*‐Lactoylglutathione	1.024	380.0962	37 087.3	0.2	[M+H]**^+^**	380.11221	C_13_H_21_N_3_O_8_S	260.0516	[85, 86]
24	l‐β‐Homoproline	1.248	130.0505	8256.4	−2.5	[M+H]**^+^**	130.08626	C_6_H_11_NO_2_	56.0494 84.0450	[87]
25	Pipecolate	1.309	130.0862	55 965.4	−1.6	[M+H]**^+^**	130.08626	C_6_H_11_NO_2_	84.0845	[88] MassBank
26	4‐Methyl‐5‐thiazole ethanol	1.647	144.1015	167 217.7	0.2	[M+H]**^+^**	144.04776	C_6_H_9_NOS	58.0648 84.0802	[89]

#### 2.4.1 Identification of Organic Acids and Derivatives

Succinic acid (1.008 min) was identified based on its [M─H]^−^ ion at *m*/*z* 117.0199 corresponding molecular formula C_4_H_6_O_4_ and the resulting fragments ions at *m*/*z* 55.0201 [M─CO_2_─H_2_O]^−^, 73.0301 [M─CO_2_]^−^ and 99.0081 [M─H_2_O]^−^. These findings are consistent with the data reported in the literature [48, 49]. To the best of our knowledge, this is the first time that succinic acid has been reported in the *Marrubium* genus.

Malic acid (1.046 min) was identified based on its [M−H]^−^ ion at *m*/*z* 133.0128 matching C_4_H_6_O_5_ Bursal et al. as well as the resulting product ions at *m*/*z* 115.0024, 89.0231, and 71.0134 corresponding to [M─H─H_2_O]^−^, [M─H─CO_2_]^−^ and [M─H─H_2_O─CO_2_]^−^, respectively [90]. This is in excellent agreement with the previous reports [50, 52]. Malic acid is found to be rich in *Marrubium astracanicum* subsp. *macrodon* [90]. Citraconic acid (RT = 1.474 min) was identified based on its deprotonated precursor [M−H]^−^ at *m*/*z* 128.96 matching C_5_H_6_O_4_ and a base peak at *m*/*z* 84.98897 corresponding to [M─H─CO_2_]^−^. The fragmentation pattern matches the description of citraconic acid as documented in the MassBank database.

#### 2.4.2 Identification of Phenolic Acids

In the present study, only two phenolic acids were identified in the residual aqueous fraction classified as hydroxycinnamic acids. Caffeic acid, also known as 3,4‐dihydroxycinnamic acid (RT = 1.087 min), was characterized in a negative mode based on its [M─H]^−^ at *m*/*z* 179.0575 fitting C_9_H_8_O_4_ [49, 55, 57]. It was also confirmed with an MS/MS spectrum showing characteristic fragment ions resulting from continuous losses of water, CO, and CO_2_ moieties at *m*/*z* 161.0459 [M─H_2_O]^−^, 143.0354 [M─H─2H_2_O]−, 135 [M─H─CO_2_]^−^, 99.0069 [M─H─2H_2_O─CO_2_]^−^, and 89.0326 [M─H─CO_2_─CO─H_2_O]^−^. This fragmentation pattern aligned with previous studies [53, 54, 56, 91]. Another fragment at *m*/*z* 71.0128 corresponds to the ion [M─H─C_6_H_4_O_2_]^‐^. Caffeic acid has previously been identified in MV [31, 43, 92, 93] and other *Marrubium* species such as *Marrubium trachyticum* Boiss [94], *Marrubium peregrinum* L., and *Marrubium friwaldskyanum* Boiss [95].

Chlorogenic acid, also known as 5‐caffeoylquinic acid (RT = 6.087 min) was identified in a positive mode based on its protonated molecular ion [M+H]^+^ at *m*/*z* 355.1042 and fitting C_9_H_8_O_4_ [58, 59, 61]. Other characteristic peaks were detected at *m*/*z* 163.0403, 145.0298, 135.0466, and 117.0314 [58, 59]. The fragment ion at *m*/*z* 163.0387 may be due to the loss of the C_7_H_11_O_6_ moiety resulting from the cleavage of the caffeoyl bond followed by the neutral loss of a water molecule [59, 60]. The fragment ions at 145.0298 and 135.0466 are obtained from the neutral loss of H_2_O and CO from the ion at *m*/*z* 163.0387, respectively. Both ions yielded the fragment at *m*/*z* 117.0314 by neutral loss of H_2_O or CO, as suggested by Willems et al. [59]. Chlorogenic acid was identified in MV [20, 96] and other *Marrubium* species [97] *Marrubium lutescens* and *M. trachyticum* Boiss [94, 98]. Caffeic acid and chlorogenic acid are common hydroxy derivatives of cinnamic acid [99]. Both acids are recognized for their high therapeutic potential due to their antioxidant, anticancer, antimicrobial, and many other properties [85, 100, 101, 102, 103].

#### 2.4.3 Identification of Flavonoids

In the current study, three subclasses of flavonoids in the residual aqueous fraction were identified. These subclasses include three flavonols, two flavones, and one flavanone, all of which are O‐ and C‐glycosides.

The compound eluted at 1.309 min showed a parent ion [M−H]^−^ at *m*/*z* 447.1173. It was tentatively identified as quercitrin (quercetin 3‐rhamnoside) matching C_21_H_20_O_11_ [62]. The peaks at *m*/*z* 149 and 151 were obtained from the retro‐Diels Alder of the aglycone quercetin and corresponded to ^1,3^B^−^ and ^1,3^A^−^ ions, respectively. Many quercetin O‐glycosides were identified in some *Marrubium* species such as quercitrin‐O‐gallate, quercetin‐7‐*O‐*galloyl‐glucoside, and quercetin‐diglucuronide in *Marrubium anisodon* as well as quercetin‐3‐*O*‐glucoside‐6″‐acetate, quercetin‐3‐*O‐*pentosyl‐pentoside, quercetin‐3‐*O‐*glucuronide and quercetin‐3‐*O*‐glucoside6″‐acetate in *Marrubium crassidens* [104]. Ibrahim et al. [105] reviewed that quercetin 3‐*O*‐β‐d‐rutinoside, quercetin 3‐*O*‐β‐d‐glucoside, quercetin 3‐*O*‐α‐1‐rhamnosyl‐glucoside, and the aglycone quercetin were identified in MV but it seems that quercitrin was identified for the first time in this species. Chen et al. reviewed the wide spectrum of pharmacological activities exhibited by quercitrin, which include reducing oxidative stress and inflammation and inhibiting microorganisms [106]. In addition, quercitrin has been shown to protect vital organs and demonstrate therapeutic potential for various diseases such as gastric ulcers, immune diseases, and cancer.

Kaempferol‐3‐glucuronide (RT = 6.251 min) was identified due to its parent ion [M−H]^−^ at *m*/*z* 461.0727 corresponding to C_21_H_18_O_12_ [62, 64], which generated a predominant daughter ion at *m*/*z* 285.0406 indicating the loss of a glucuronic acid residue (176 Da) [65]. Another peak at *m*/*z* 175 was observed and explained by the loss of an aglycone portion [63]. In the positive mode, this compound was eluted at 9.756 min and showed a parent ion [M + H]^+^ at *m*/*z* 463.0873, which generated an intense daughter ion at *m*/*z* 287.0552 relative to the ion [M + H−176]^+^ [66]. Kaempferol‐3‐glucuronide was identified in *M. crassidens* [104]. It was reported that kaempferol‐3‐glucuronide has shown high scavenging activity and effectiveness in treating various types of cancers [51, 107].

Isorhamnetin‐3‐*O*‐rutinoside (RT = 7.484 min) was identified as it yielded a negative precursor *m*/*z* = 623.1998 [M−H]^−^ corresponding molecular formula C_28_H_32_O_16_ [72], which is confirmed by the detection of fragment ions at *m*/*z* values 461.1672 [M−H−162]^−^ and 315.1061 [M−H−162−146]^−^ corresponding to the aglycone part (isorhamnetin). The total loss of 308 Da is characteristic of the disaccharide moiety (rutinose) [65, 67]. This compound was identified in *Marrubium globosum* [105] and *M. friwaldskyanum* [95]. As reported in previous research papers, it was found that isorhamnetin‐3‐*O*‐rutinoside exhibited high scavenging and antiproliferative effects [108, 109].

The compound eluted at 5.785 min gave an [M−H]^−^ ion at *m*/*z* 593.1507 in negative mode, and in positive mode, it eluted at 6.117 min and yielded an [M + H]^+^ ion at *m*/*z* 595.1673 indicating a molecular formula of C_27_H_30_O_15_, was identified as vicenin‐2 also known as apigenin 6,8‐di‐C‐glucoside. The fragmentation pattern aligns with the description of vicenin‐2 [70, 73, 74, 110]. On the other hand, the fragmentation pattern of the protonated vicenin‐2 ion [M+H]^+^ is complex. Abundant water‐loss ions at *m*/*z* 577 ([M+H−H_2_O]^+^), *m*/*z* 559 [M + H−2H_2_O]^+^, and *m*/*z* 529 ([M + H−CH_2_O−2H_2_O]^+^) were observed [76, 111]. In addition, other peaks derived from the loss of 120 and/or 150 Da from the protonated molecule, such as *m*/*z* 295^0,1^X^+^_1_^0,1^X^+^_2_ [M+H−150−150]^+^; *m*/*z* 325^0,1^X^+^_1_^0,2^X^+^_2_ [M+H−120−150]^+^, *m*/*z* 337^0,2^X^+^_1_^0,2^X^+^_2_−H_2_O [M+H−120−120−18]^+^; *m*/*z* 379^0,2^X^+^_1_^0,4^X^+^_2_−2H_2_O [M + H−120−96]^+^ and *m*/*z* 457^0,2^X^+^_1_−H_2_O [M + H−120−18]^+^. This fragmentation pattern is consistent with previous studies [69, 75, 76, 112]. Fragmentation sites were displayed in the supporting information Figure S4. Apigenin and some derivatives have been identified in MV as O‐glycosides, O‐glucuronides, and C‐glycosides [113, 114]. Vicenin‐2 has been noted to exhibit several pharmacological properties, including antioxidant liver‐protective, anti‐inflammatory, and anticancer effects [115].

Baicalein‐7‐*O*‐glucuronide, also known as baicalin (RT = 6.732 min) was tentatively identified based on the molecular ion [M−H]^−^ at *m*/*z* = 445.0794 fitting C_21_H_18_O_11_ along with a predominant fragment ion at *m*/*z* value 269.0476 [M−H−176]^−^ which corresponds to the loss of glucuronic acid [65, 72]. Another peak at *m*/*z* 175.0254 was observed and explained by the loss of an aglycone portion [77]. This compound was previously found in the *Marrubium* genus and recently demonstrated in MV. In a recent review, Bao et al. emphasized the health benefits of baicalin, such as its anti‐inflammatory properties, ability to reduce oxidative stress, inhibit various viruses, and potentially suppress the growth of different tumor cells [116].

Naringenin‐7‐*O* glucoside, also known as prunin (RT = 8.845 min) was identified based on its protonated molecular ion [M+H]^+^ at *m*/*z* 435.1280 which corresponds C_21_H_22_O_10_ [78]. The peak detected at 273.0377 resulted from the loss of glycoside moiety (162 Da), which is an indicator of aglycon residue (naringenin). Recently, naringenin 7‐*O*‐glucoside was identified in *Marrubium alysson* L. and *M. globosum* [88, 105]. In a recent study, naringenin‐7‐*O*‐glucoside was tested in vitro against a panel of cancer cell lines. It was demonstrated that this flavone glycoside has the potential to be a targeted therapeutic agent [117].

### 2.5 Implications of Major Compounds in Biological Activity

Among the 26 compounds identified in MVRF using UPLC‐ESI‐QTOF‐MS/MS, five major compounds were selected: malic acid, caffeic acid, chlorogenic acid, kaempferol‐3‐glucuronide, and l‐tryptophan. These compounds are known for their antioxidant activity and cytotoxic effects.

The antioxidant potential of malic acid [118, 119], caffeic acid [102, 120, 121], chlorogenic acid [121, 122], kaempferol‐3‐glucuronide (kaempferol linked to a glucuronic acid moiety via a glycosidic bond at the 3‐position of the flavonoid) [123], and l‐tryptophan [124] has been reported. In addition, the potential of malic acid to induce anticarcinogenic and genotoxic damage in human fibroblast cells (HDFa) and glioblastoma (U87‐MG) cell lines has been demonstrated [125]. The antitumor action of caffeic acid, including its inhibitory effect on cell migration and reducing metastases in tumor cells, has been investigated [126, 127]. Also, the anticancer potential of caffeic acid in several human cancers is well documented [128, 129, 130]. Chlorogenic acid has been noted for its role in cancer prevention and therapy [131], with evidence suggesting its ability to induce apoptosis and cell‐cycle arrest [132]. The biological effects of kaempferol‐3‐glucuronide, similar to kaempferol, have been observed in several cancer cell lines. In addition, the role of kaempferol in inhibiting the invasion of human breast carcinoma has been explored [133].

Based on the above literature data, malic acid, caffeic acid, chlorogenic acid, kaempferol‐3‐glucuronide, and l‐tryptophan were selected as key compounds for subsequent molecular docking calculations and pharmacokinetic profiling.

### 2.6 Molecular Docking Analysis

**FIGURE 1:**
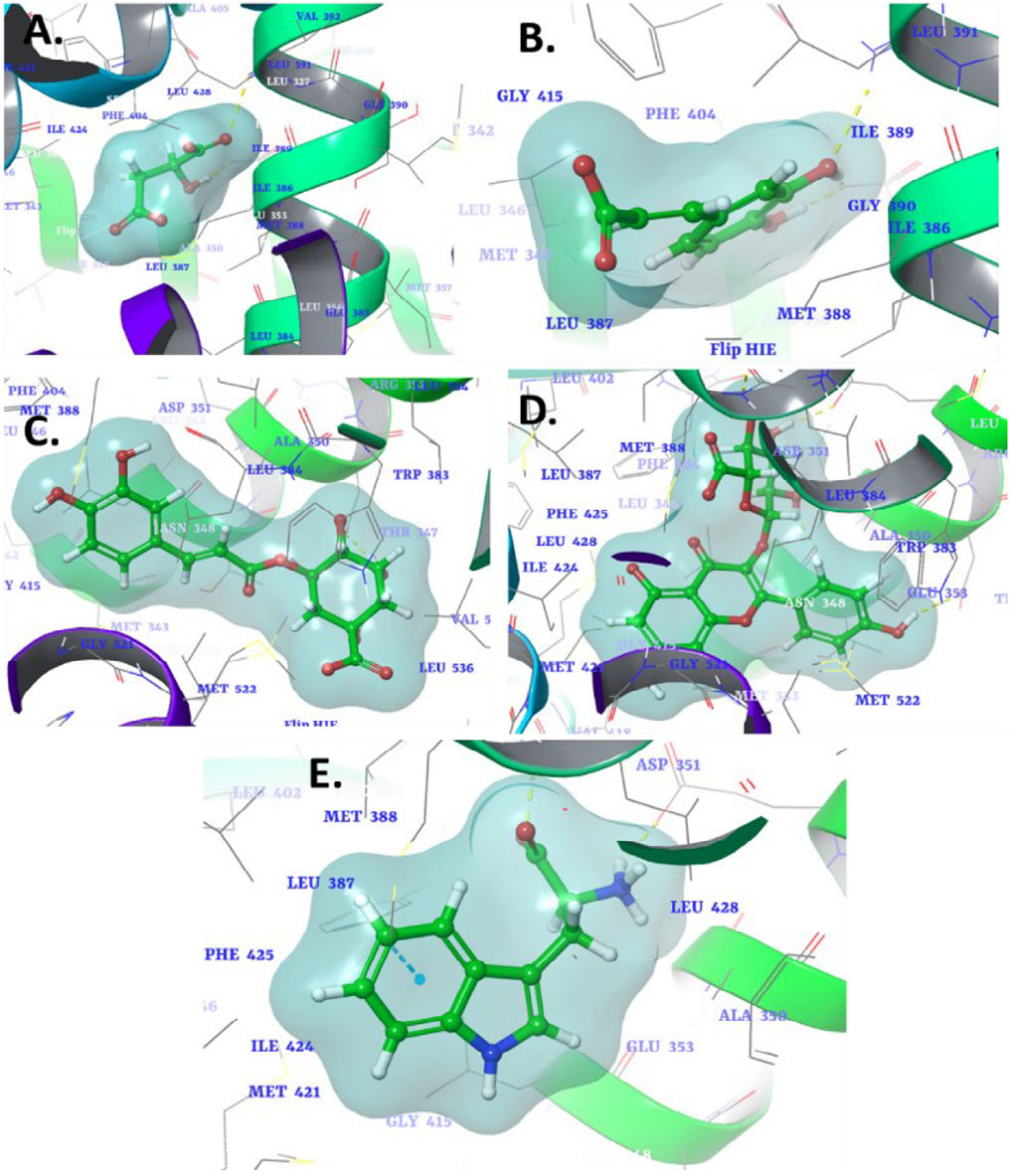
3D binding interaction diagram of phytocompound (A) malic acid, (B) caffeic acid, (C) chlorogenic acid, (D) kaempferol‐3‐glucuronide, (E) l‐tryptophan into the active sites of estrogen receptor alpha (PDB ID: 6CHZ).

Estrogen receptor alpha (ERα) is a nuclear hormone receptor activated by the hormone estrogen. It plays a crucial role in regulating the growth and development of tissues, particularly in the breast and reproductive organs. ERα is significantly involved in the pathogenesis of hormone‐driven cancers, especially BC, where its overexpression promotes tumor growth and survival [134, 135]. In contrast, human thymidylate synthase (TS) is a critical enzyme in the folate pathway, responsible for catalyzing the conversion of deoxyuridylate (dUMP) to thymidylate (dTMP), a nucleotide essential for DNA synthesis and repair. The activity of TS is particularly vital for rapidly dividing cells, such as cancer cells, which have an increased demand for DNA replication [136]. The docking results for the phytocompounds against ERα (PDB ID: 6CHZ) and TS (PDB ID: 1HVY) are summarized in Table 5, with docking scores indicating the binding affinities of each compound. In the 6CHZ protein, malic acid exhibited a binding affinity of −6.254 kcal/mol, interacting with the active site of the receptor 6CHZ through key interactions (Figure 1A). A hydrogen bond was observed with residues Glu353 and Arg394, suggesting stabilization through electrostatic interactions. In addition, Arg394 was involved in an ionic interaction, which may enhance the binding strength and contribute to the overall stability of the complex. With a binding affinity of −6.992 kcal/mol, caffeic acid demonstrated stronger interactions, particularly through hydrogen bonding. Arg394 and Glu353 were key residues forming hydrogen bonds, indicating a firm and potentially selective binding mode (Figure 1B). These interactions could facilitate the proper positioning of the compound in the binding pocket, enhancing its potential biological activity.

**TABLE 5:** Docking score (in kcal/mol) of the phytocompounds in the active site of estrogen receptor alpha (PDB ID: 6CHZ) and human thymidylate synthase (PDB ID: 1HVY).

Phytocompounds	6CHZ	1HVY
Malic acid	−6.254	−6.848
Caffeic acid	−6.992	−6.228
Chlorogenic acid	−6.164	−5.228
Kaempferol‐3‐glucuronide	−8.715	−5.66
l‐Tryptophan	−7.652	−4.157

**FIGURE 2:**
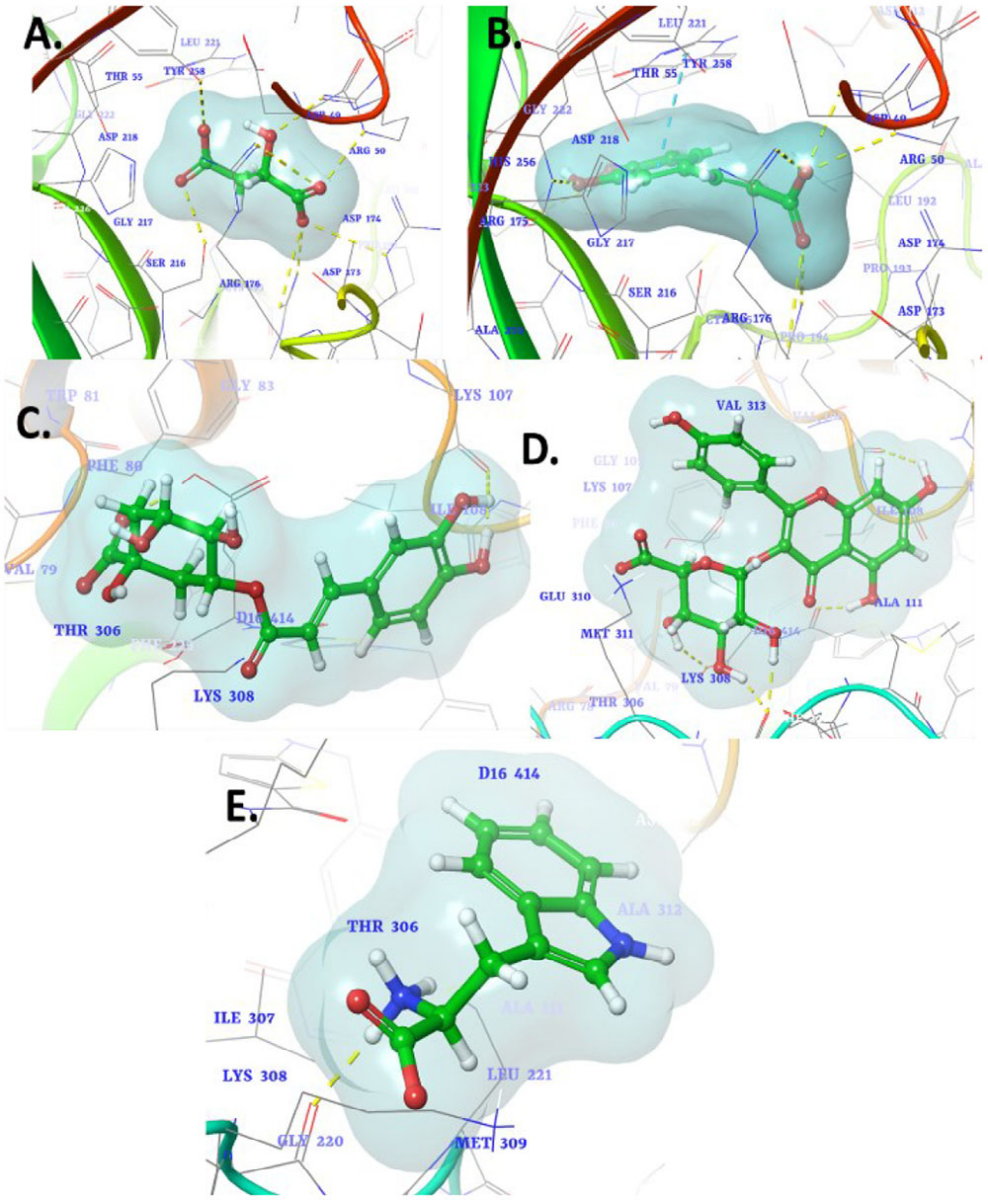
3D binding interaction diagram of phytocompound (A) malic acid, (B) caffeic acid, (C) chlorogenic acid, (D) kaempferol‐3‐glucuronide, (E) l‐tryptophan into the active sites of human thymidylate synthase (PDB ID: 1HVY).

Chlorogenic acid displayed a moderate binding affinity of −6.164 kcal/mol, forming a critical interaction with Asp351 (Figure 2C). This single interaction may suggest a less robust binding compared to other compounds, but its affinity could still contribute to significant biological activity, depending on the overall dynamics of the binding site environment. Kaempferol‐3‐glucuronide emerged as the most potent among the compounds, with a binding affinity of −8.715 kcal/mol. The compound exhibited multiple interactions with key residues, including Leu387, Glu353, Asp351, and Leu346 (Figure 1D). This multitude of interactions suggests strong and stable binding, likely due to the complementarity between the molecule and the receptor pocket. The involvement of both hydrogen bonds and hydrophobic contact could explain the high binding affinity observed. l‐Tryptophan displayed a binding affinity of −7.652 kcal/mol and stood out due to its unique π–π interaction with Phe404, suggesting a key role for aromatic stacking in stabilizing the compound within the binding site (Figure 1E). In addition, ionic interactions with Arg394 and Glu353 further stabilized the complex, reinforcing the versatility of l‐tryptophan in engaging with multiple interaction types within the binding site. The co‐crystallized ligand (H3B‐9224) in this protein exhibited critical hydrogen bonding interactions with Glu353 and Arg394, which are key residues within the active site [137]. These interactions play a vital role in stabilizing the ligand and facilitating its optimal orientation for effective binding. A similar binding pattern was observed with the identified phytochemicals, including hydrogen bonding with these pivotal residues. This similarity suggests that the phytochemicals mimic the binding mechanism of the co‐crystallized ligand. The observed interactions highlight the structural and chemical compatibility of the phytochemicals with the binding site, reinforcing their potential as effective inhibitors of the 6CHZ protein.

The molecular docking study of selected phytocompounds against the protein target (PDB ID: 1HVY) revealed varying binding affinities and interactions with key residues. Among the tested compounds, malic acid demonstrated the highest binding affinity with a docking score of −6.848 kcal/mol. It exhibited multiple interactions, including hydrogen bonds with Tyr258, Ser216, and Arg50, and ionic interactions with Arg176, Arg215, and Arg50, suggesting a strong and stable binding to the active site (Figure 2A). Caffeic acid, with a docking score of −6.228 kcal/mol, showed hydrogen bonding with Asp218, Asn226, and Arg215, as well as ionic interactions with Arg50, Arg175, and Arg176. In addition, a π–π interaction was observed with Ala414, further stabilizing its binding within the active site (Figure 2B). Chlorogenic acid had a lower binding affinity (−5.228 kcal/mol), forming a single hydrogen bond with Lys107 (Figure 2C). Kaempferol‐3‐glucuronide, with a docking score of −5.66 kcal/mol, interacted through hydrogen bonding with Lys107 and Met309, indicating moderate binding strength (Figure 2D). l‐Tryptophan, which exhibited the lowest binding affinity (−4.157 kcal/mol), formed hydrogen bonds with Met309 and ionic interactions with Lys308 (Figure 2E). While the binding strength was relatively weaker, these interactions suggest their potential for stabilization within the active site. The co‐crystallized ligand raltitrexed, a well‐known inhibitor, served as a benchmark for evaluating the binding interactions of the tested phytochemicals. Raltitrexed exhibited critical interactions within the active site of the target protein, including ionic interactions with Lys308, which play a pivotal role in stabilizing its binding conformation [138]. Furthermore, it established a hydrogen bond with Lys77, contributing to its anchoring within the binding pocket and enhancing its specificity towards the target. In addition, a salt bridge interaction with Lys308 was observed, reinforcing the ligand's overall stability and interaction strength. Overall, the results indicate that malic acid and caffeic acid have the strongest binding interactions with the target protein, primarily through hydrogen bonding and ionic interactions with critical residues, making them promising candidates for further investigation.

### 2.7 Pharmacokinetic and Drug‐Likeness Properties

As a preliminary step in drug discovery research and to forecast insights into their potential use as therapeutic agents, the selected phytoconstituents (Table 6) in MVRF were tested for their pharmacokinetic properties based on their absorption, distribution, metabolism, excretion, and toxicity (ADMET) profile [139, 140, 141, 142, 143]. Predicted values of Caco‐2 permeability indicate moderate to lower absorption. It was predicted that compounds **2**, **3**, and **5** would exhibit good intestinal absorption (in humans), with values exceeding 30%. With regard to the permeability of the selected phytoconstituents across the skin, all exhibited low permeability. The fraction unbound, which delineates the extravasate portion of the free drug in plasma, is within the range of 0.28 and 0.568, indicating that the drug exhibited a favorable distribution profile in the plasma and primarily interacted with the pharmacological target.

**TABLE 6:** Pharmacokinetic profile assessment of major MVRF phytoconstituents.

Entry	1	2	3	4	5	Reference
Absorption						
Water solubility	−1.381	−2.33	−2.449	−2.866	−2.891	—
Caco‐2 permeability	−0.395	0.634	−0.84	−0.884	0.638	> 0.9
Intestinal absorption (human)	13.831	69.407	36.377	25.165	77.224	< 30% is poorly
Skin permeability (log Kp)	−2.735	−2.722	−2.735	−2.735	−2.735	> −2.5 is low
P‐glycoprotein inhibitor	No	No	Yes	Yes	Yes	No
Distribution						
VDss (human)	−0.998	−1.098	0.581	1.295	−0.081	Low is < −0.15, high is > 0.45
Fraction unbound (human)	0.652	0.529	0.658	0.28	0.577	—
BBB permeability	−0.788	−0.647	−1.407	−1.441	−0.495	Poorly is < −1, high is > 0.3
CNS permeability	−3.523	−2.608	−3.856	−3.955	−2.622	Penetrate is > −2, unable is < −3
Metabolism						
CYP1A2 inhibitior	No	No	No	No	No	No
CYP2C19 inhibitior	No	No	No	No	No	No
CYP2C9 inhibitior	No	No	No	No	No	No
CYP2D6 inhibitior	No	No	No	No	No	No
CYP3A4 inhibitior	No	No	No	No	No	No
Excretion						
Total clearance	0.81	0.508	0.307	0.503	0.64	—
Renal OCT2 substrate	No	No	No	No	No	—
Toxicity						
AMES toxicity	No	No	No	No	No	No
Max. tolerated dose (human)	1.212	1.145	−0.134	0.46	0.793	Low is ≤ 0.477, high is > 0.477
hERG I inhibitor	No	No	No	No	No	No
hERG II inhibitor	No	No	No	No	No	No
Oral rat acute toxicity (LD_50_)	1.818	2.383	1.973	2.513	2.357	—
Oral rat chronic toxicity (LOAEL)	3.104	2.092	2.982	4.641	1.231	—
Hepatotoxicity	No	No	No	No	No	No
Skin sensitization	No	No	No	No	No	No
*Tetrahymena pyriformis* toxicity	0.285	0.293	0.285	0.285	0.285	—
Minnow toxicity	3.348	2.246	5.741	6.898	1.835	—

*Note*: **1**, malic acid; **2**, caffeic acid; **3**, chlorogenic acid; **4**, kaempferol‐3‐glucuronide; **5**: l‐tryptophan.

The volume of distribution (VDss) is a measure of the extent of drug distribution. The values of compounds **3** and **4** indicate that they will be distributed in tissue, whereas those of compounds **1** and **2** suggest that they will be distributed in plasma. With regard to blood–brain barrier (BBB) membrane permeability, compounds **3** and **4** exhibited a logBB value of less than −1, indicating that they were predicted to have difficulty crossing the BBB. In contrast, compounds **1**, **2**, and **5** demonstrated the ability to cross the BBB to a moderate extent. It is similarly postulated that compounds **2** and **5** will penetrate the central nervous system (CNS) with ease. The drug's metabolic profile was predicted using cytochrome P450s, with the two main subtypes being CYP2D6 and CYP3A4 enzymes. The generated values indicated that none of the selected phytoconstituents exhibited inhibitory effects on any P450 isoform. The capacity of the body to eliminate the drug was determined by the total clearance, which encompasses both hepatic and renal processes. As illustrated in Table 6, our findings indicated that only compound **6** exhibited effective elimination. A toxicity assessment was also conducted for the selected phytoconstituents. The toxicity results were found to be promising, with none of the selected compounds exhibiting AMES, hepatotoxicity, or skin sensitization.

### 2.8 Target Prediction

**FIGURE 3:**
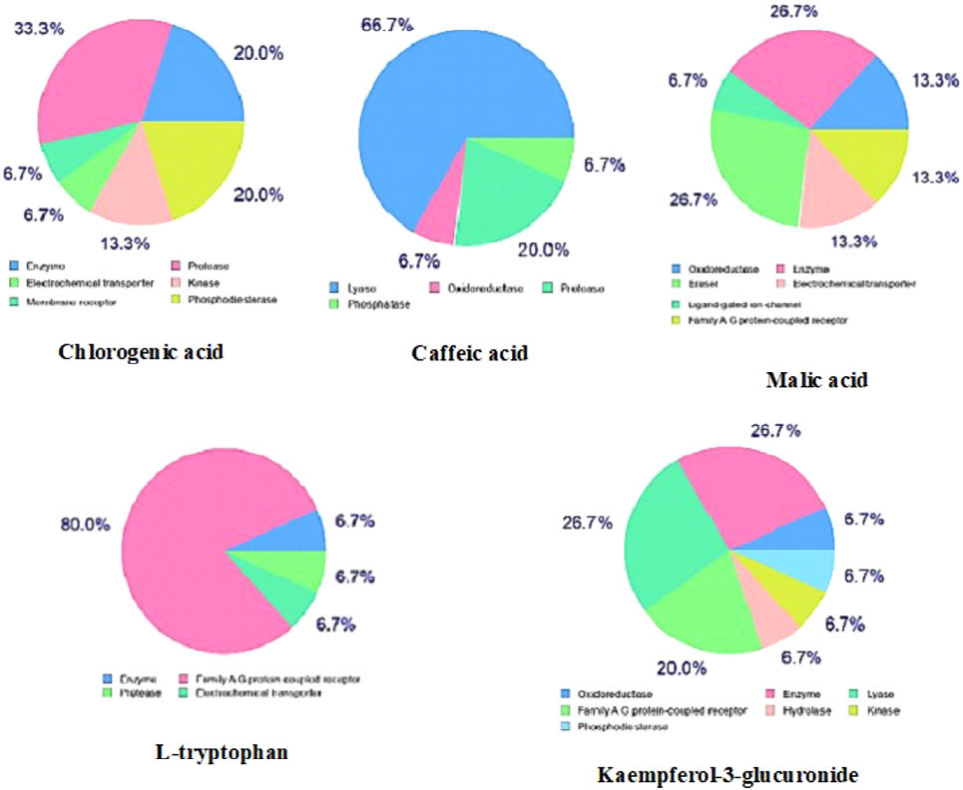
Biological target types likely affected by the key phytochemicals in MVRF.

The potential molecular targets for the major phytochemicals (chlorogenic acid, caffeic acid, malic acid, l‐tryptophan, and kaempferol‐3‐glucuronide) were predicted and the results revealed that they are categorized into several classes. As illustrated in Figure 3, the predominant target class for malic acid was enzyme (26.7%), for caffeic acid was lyase (66.7%), for chlorogenic acid was protease (33.3%), for kaempferol‐3‐glucuronide was enzyme (26.7%) and family AG protein‐coupled receptor (26.7%), and for l‐tryptophan was family AG protein‐coupled receptor (80%).

## 3 Conclusions

The extracts evaluated demonstrated promising potential as sources of antioxidant and anticancer compounds. The antioxidant activity, as demonstrated by the DPPH^•^ and ABTS^+•^ radical scavenging assays, exhibited significant cellular toxicity against the MCF7 (breast), HT29 (colon), and SW480 (colorectal) cell lines. This observation indicates a direct correlation between the observed biological effects and the presence of phenolic compounds. UPLC‐QTOF‐MS/MS analysis identified a variety of bioactive metabolites, including three organic acids, two phenolic acids, three flavonols, two flavones, one flavanone, five amino acids, one bile acid, and one triterpene saponin. In addition to these, other compounds were also identified and are considered coadjuvants in the observed biological activities. In silico analyses corroborated the in vitro findings, thereby reinforcing the therapeutic potential of the MVRF. However, for these results to be safely translated into clinical application, additional in vivo studies and clinical trials are needed to identify the bioactive compounds responsible for the observed effects and validate their efficacy and safety in humans.

## 4 Experimental Section

### 4.1 Chemical and Reagents

The HPLC‐grade methanol, formic acid (≥ 98%) and sodium hydroxide anhydrous pellets were sourced from Fisher Scientific (UK). Ammonium formate (mass spectrometer grade, 98% or greater) and HPLC‐grade acetonitrile were obtained from Sigma‐Aldrich (Germany). Ultrapure water for chromatographic purposes was prepared using a purification system (Millipore Milli‐Q, USA). The remaining chemicals and reagents were of analytical grade.

### 4.2 Plant Collection and Preparation of Extracts

#### 4.2.1 Plant Collection

**FIGURE 4:**
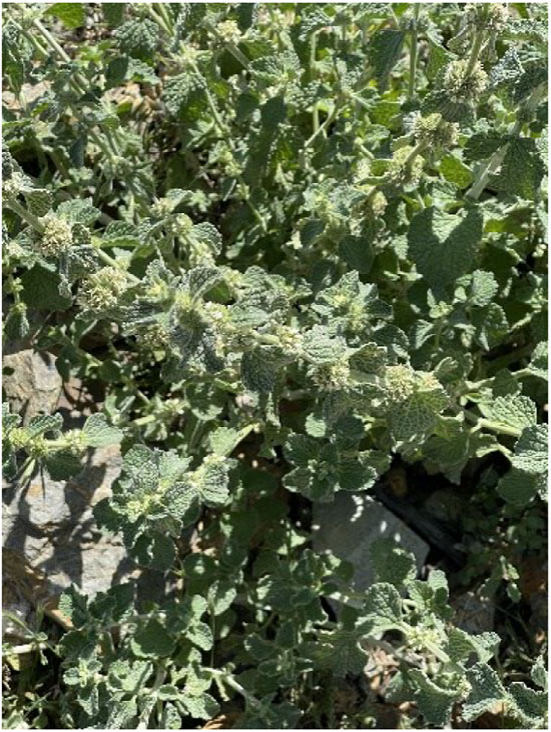
Illustration of aerial parts of *Marrubium vulgare* L.

In April 2022, aerial parts of MV were collected (Figure 4). The plants were harvested from Buljurashi (Al‐Baha Province) in southern Saudi Arabia (coordinates 41.58′ N and 19.86′ E, altitude 2450 m) at the flowering stage. The plant material was authenticated by an expert taxonomist, Dr. Haidar A. Mohamed of the Department of Biology, Faculty of Science, Al‐Baha University. The available databases were consulted for the purpose of verifying the nomenclature [144, 145]. The plant was archived with voucher specimen herbarium number BUH‐112 at the Pharmacognosy Department, Faculty of Clinical Pharmacy, Al‐Baha University, KSA.

#### 4.2.2 Preparation of Extracts

The aerial parts of MV (500 g) were dried in a shaded environment, ground, and then successively extracted at room temperature with ethanol and water (8:2, 3 × 1.5 L), yielding 14% of the MVCE. The residue was subjected to fractionation, resulting in the isolation of five distinct fractions: petroleum ether (MVPF, 4.1 g), chloroform (MVCF, 12.9 g), ethyl acetate (MVEF, 1.3 g), and an aqueous residual fraction (MVRF, 10.9 g).

### 4.3 Phytochemical Screening, TPC, and TFC

A preliminary qualitative phytochemical screening was conducted to identify the phytochemical constituents of the crude extract of MV aerial parts using standard protocols [146]. The following phytochemicals were subjected to analysis: triterpenes, alkaloids, saponins, cardiac glycosides, steroids, flavonoids, tannins, and phenolic compounds.

The TPC of the crude extract (MVCE) was quantified in accordance with the methodology established by Pourmorad et al. [147] and expressed as mg GAE/g DW.

The TFC was also determined based on the method described by Pourmorad et al. [147], with some modifications, and was expressed in mg RE/g DW.

### 4.4 Antioxidant Activity

#### 4.4.1 DPPH^•^ and ABTS^+•^ Radical Scavenging Activities

The method for determining antioxidant activity based on the free radical DPPH**^•^** (2,2‐diphenyl‐1‐picrylhydrazyl) was performed according to procedures previously described by [148]. The technique involves reducing the DPPH**^•^** radical in a methanolic solution in the presence of antioxidant compounds, which is indicated by a decrease in absorbance at 517 nm. Briefly, a DPPH**^•^** solution (2 mL, 0.1 mM) was prepared in methanol and mixed with 1 mL of various concentrations of MV extracts or the antioxidant standard compound (Trolox). The mixture was incubated at room temperature, in the dark, for 1 h. After this period, the absorbance was measured in a spectrophotometer. The percentage of inhibition of the DPPH**^•^** radical was calculated using the following Equation (1).

(1) $$\mathrm{Inhibition}\;\left(\%\right)=\frac{\mathrm{Control}\;\mathrm{absorbance}\;-\;\mathrm{Sample}\;\mathrm{absorbance}}{\mathrm{Control}\;\mathrm{absorbance}}\times 100$$

The results were expressed in terms of IC_50_ (concentration required to inhibit 50% of DPPH**^•^**), calculated by linear fitting of the tested concentrations.

The method for determining antioxidant activity based on the ABTS^•+^ radical cation discoloration assay was performed according to methods described in the literature [149]. Briefly, the ABTS^•+^ cation radical was generated by the reaction of an aqueous solution of ABTS (7 mM) with potassium persulfate (2.45 mM) and the mixture was kept in the dark at room temperature for 12–16 h before use. The formed ABTS^•+^ radical cation was diluted in ethanol to an absorbance of 0.700 ± 0.020 at 734 nm. The antioxidant activity was assessed by adding 10 µL of the sample or antioxidant standard to 1 mL of the ABTS^•+^ solution. The absorbance measurement was then performed after 30 min at 30°C. Appropriate solvents were used as controls in each assay. All determinations were carried out in triplicate. The percentage inhibition of the ABTS^•+^ radical was calculated using the following Equation (1). The results were expressed as Trolox‐equivalent antioxidant activity (TEAC), calculated in relation to a standard curve constructed with different concentrations of Trolox and IC_50_.

### 4.5 Cytotoxicity

#### 4.5.1 Cell Lines

This study employed three cancer cell lines: MCF7 (human breast adenocarcinoma), HT29, and SW480 (human colorectal adenocarcinoma). Furthermore, MRC5 (normal human fetal lung fibroblast) cells were procured from the ATCC (USA). The three cancer cells were subcultured in RPMI‐1640 media in accordance with the methodology described by Abdalla et al. [150].

#### 4.5.2 The Cytotoxicity Determination

The cytotoxicity of the six extracts, along with DOX, was evaluated using the MTT assay, as previously described [151].

### 4.6 Phytochemical Profile by UPLC‐ESI‐QTOF‐MS/MS Analysis

The UPLC‐ESI‐QTOF‐MS/MS analysis was conducted according to Mohammed et al. [72].

#### 4.6.1 Sample Preparation

A stock solution of the extract was created using 50 mg of the lyophilized aqueous ethanolic extract, which was dissolved in 1000 µL of a solvent blend comprising water, methanol, and acetonitrile (H_2_O:MeOH:ACN) at a ratio of 2:1:1. The stock solution was rendered fully soluble by vortexing the sample and subjecting it to ultrasonication at 30 kHz for a period of 10 min. A 20 µL aliquot of the stock solution was diluted once more with 1000 µL of H_2_O:MeOH:ACN (2:1:1) and subjected to centrifugation at 10 000 rpm for 5 min. The resulting solution was then injected for analysis at a concentration of 1 µg/mL. Similarly, an LC‐MS analysis was conducted for the blank and quality control samples/internal standard (IS) to ensure the reliability of the experiment. The sample was introduced in both positive and negative modes.

#### 4.6.2 Instruments and Acquisition Method

The isolation of small molecules was conducted using an ExionLC system (AB Sciex, Framingham, MA, USA), which was linked to an autosampler, an in‐line filter disk pre‐column (0.5 µm × 3.0 mm, Phenomenex, Torrance, CA, USA), and an Xbridge C_18_ column (3.5 µm, 2.1 × 50 mm) (Waters Corporation, Milford, MA, USA). The column was maintained at 40°C and operated at a flow rate of 300 µL/min. The mobile phase consisted of Solution A, which was 5 mM ammonium formate in 1% methanol, with the pH adjusted to 3.0 using formic acid, and Solution B, which was acetonitrile (100%) for the positive mode. The negative mode solution (C) consisted of 5 mM ammonium formate in 1% methanol, modified to pH 8 with sodium hydroxide. The gradient elution was conducted according to the following schedule: The initial phase of the elution process was conducted for 20 min at a concentration of 10% B, followed by a 5‐min period at 90% B. Subsequently, the concentration was reduced to 10% B for a further 5 min, after which the process was terminated, and the column was allowed to undergo a period of equilibration at 90% B.

The MS analysis was conducted using a Triple TOF 5600+ system, which is equipped with a Duo‐Spray source operating in ESI mode (AB SCIEX, Concord, Ontario, Canada). The sprayer capillary and declustering potential voltages were set to 4500 and 80 eV in positive mode and −4500 and −80 V in negative mode. The source temperature was set at 600°C, the curtain gas at 25 psi, and both Gas 1 and Gas 2 at 40 psi. The collision energy was set at 35 V (positive mode) and −35 V (negative mode), with a CE spread of 20 V and an ion tolerance of 10 ppm. The TripleTOF 5600+ was operated with an information‐dependent acquisition (IDA) protocol. Batches for the collection of MS and MS/MS data were generated with the use of Analyst‐TF 1.7.1. The IDA technique was employed to simultaneously gather full‐scan MS and MS/MS data. The methodology entailed the acquisition of high‐resolution survey spectra spanning a mass range of 50–1100 *m*/*z*, with the mass spectrometer employed in the aforementioned configuration [152].

#### 4.6.3 LC‐MS Data Processing

The open‐source software MS‐DIAL 3.70 [153] was employed for a comprehensive analysis of small molecules in the absence of any targeted sampling. The ReSpect positive database (2737 records) and the ReSpect negative database (1573 records) were selected as reference databases based on the acquisition method. The search settings were established with MS1 and MS2 mass tolerance at 0.01 and 0.05 Da for data acquisition, with a minimum peak height of 100 amplitude, a mass slice width of 0.05 Da, a smoothing level of two scans, and a minimum peak width of six scans. For identification, the MS1 and MS2 tolerances were set as follows: The alignment tolerance was set at 0.2 Da, while the retention time tolerance was 0.05 min, and the MS1 tolerance was set at 0.25 Da. The MS‐DIAL results were employed to reprocess the data in PeakView 2.2 with the MasterView 1.1 package (AB SCIEX) for the purpose of confirming features (peaks) from the TIC in accordance with the following criteria: aligned features with a signal‐to‐noise ratio exceeding 5 and sample intensities relative to the blank exceeding 5.

### 4.7 In Silico Molecular Docking

The structures of the phytocompounds were obtained from PubChem and processed using Schrödinger's LigPrep, which optimizes ligand geometry and generates low‐energy 3D structures with accurate chirality. Molecular docking [154] of the phytocompounds was performed using Schrödinger's Glide into the active sites of ERα (PDB ID: 6CHZ) and human TS (PDB ID: 1HVY). The protein crystal structures were preprocessed as follows [155, 156]. Finally, docking was conducted in Glide using the standard precision (SP) method [157].

### 4.8 Pharmacokinetics Study

In silico molecular studies of the selected phytoconstituents were investigated to predict their pharmacokinetic (https://biosig.lab.uq.edu.au/pkcsm/) and target prediction (http://www.swisstargetprediction.ch/) characteristics.

### 4.9 Statistical Analysis

All assays were conducted in triplicate, and the data was expressed as mean ± standard deviation (SD). Differences between the data were analyzed using Tukey's one‐way analysis of variance (ANOVA) with SPSS software (version 22.0).

## Author Contributions

**Ines El Mannoubi**: led the conception and design of the study, supervised the execution of the research, and coordinated the writing of the manuscript. **Mozaniel Santana de Oliveira**: contributed significantly to the methodological structuring and data analysis. **Nuha M. Alghamdi**: Contributed to data collection and analysis. **Seham H. Bashir**: contributed to data collection and analysis. **Suada Alsaied Mohamed**: contributed to data collection and analysis. **Hedia Chaabane**: participated in the interpretation of results and critical review of the manuscript. **Ashraf N. Abdalla**: participated in the interpretation of results and critical review of the manuscript. **Majdi Abid**: participated in the interpretation of results and critical review of the manuscript. **Adel Kadri**: coordinated the writing of the manuscript, provided technical support and academic supervision.

## Conflicts of Interest

The authors declare no conflicts of interest.

## Supporting information




**Supporting Fig. S1**: Total ion chromatogram (TIC) of MVRF in (a) ESI^−^ mode, (b) ESI^+^ mode. **Supporting Fig. S2**: MS/MS spectra and fragmentation patterns of some identified secondary metabolites in MVRF. **Supporting Fig. S3**: Structure of identified phenolic compouds in RFMV by UPLC‐ESI‐QTOF‐MS/MS. **Supporting Fig. S4**: Fragmentation sites of vicenin‐2 at C‐glycosidic hexose moieties.

## Data Availability

The data that support the findings of this study are available from the corresponding author upon reasonable request.
