# Supplementary material for: UPLC‐ESI‐QTOF‐MS/MS Profiling, Antioxidant, and Cytotoxicity Potentials of Marrubium vulgare L. Extracts: Experimental Analysis and Computational Validation
Source: Chem Biodivers. 2025 Jun 14;22(10):e00400. doi: 10.1002/cbdv.202500400 (PMC12533807; doi:10.1002/cbdv.202500400)
Supplement: Supplementary file 1 — Supporting Fig. S1: Total ion chromatogram (TIC) of MVRF in (a) ESI− mode, (b) ESI+ mode. Supporting Fig. S2: MS/MS spectra and fragmentation patterns of some identified secondary metabolites in MVRF. Supporting Fig. S3: Structure of identified phenolic compouds in RFMV by UPLC‐ESI‐QTOF‐MS/MS. Supporting Fig. S4: Fragmentation sites of vicenin‐2 at C‐glycosidic hexose moieties. [file CBDV-22-e00400-s001.pdf]

## Supporting Information

# UPLC-ESI-QTOF-MS/MS Profiling, Antioxidant, and Cytotoxicity Potentials of *Marrubium vulgare* L. Extracts: Experimental Analysis and Computational Validation

Ines El Mannoubi[a,b]*, Nuha M. Alghamdi[a], Seham H. Bashir[a,c], Suada Alsaied Mohamed[a], Hedia Chaabane[d], Ashraf N. Abdalla[e,f], Majdi Abid[g], Adel Kadri[a,h], Mozaniel Santana de Oliveira[i]*

[a] Chemistry Department, Faculty of Science, Al-Baha University, Kingdom of Saudi Arabia
[b] Research Unit Advanced Materials, Applied Mechanics, Innovative Processes, and Environment, UR22ES04, Higher Institute of Applied Sciences and Technology of Gabes, University of Gabes, 6072, Tunisia
[c] Department of Chemistry and Industrial Chemistry, College of Applied and Industrial Sciences, University of Bahri, Sudan
[d] Laboratory of Natural Substances, National Institute of Research and Physico-Chemical Analysis, Ariana, Tunisia
[e] Department of Pharmacology and Toxicology, College of Pharmacy, Umm Al-Qura University, Makkah 21955, Saudi Arabia
[f] Department of Pharmacology and Toxicology, Medicinal and Aromatic Plants Research Institute, National Center for Research, Khartoum 2424, Sudan
[g] Department of Chemistry, College of Science, Jouf University, P.O. Box 2014, Sakaka, Aljouf, Kingdom of Saudi Arabia
[h] Faculty of Science of Sfax, Department of Chemistry, University of Sfax, B.P. 1171, 3000 Sfax, Tunisia.
[i] Postgraduate Program in Pharmaceutical Sciences (PPGCF), Institute of Health Sciences, Federal University of Pará, R. Augusto Corrêa, 01-66075-110 Guamá, Belém – Brazil

***Corresponding Author**: Ines El Mannoubi, ielmannoubi@bu.edu.sa, and Mozaniel Santana de Oliveira mozaniel.oliveira@yahoo.com.br

Abstract: Plant extracts are emerging as valuable options for food additives and therapeutic treatments. This study evaluated the phytochemical profile, antioxidant activity, and cytotoxicity of aerial parts of *Marrubium vulgare* L. crude extract (MVCE) and its subfractions. The MVCE (80% ethanol) contained steroids, phenolic compounds, flavonoids, terpenes, and cardiac glycosides, with total phenolic content (TPC) and total flavonoid content (TFC) of 14.96 ± 0.12 mg GAE/g DW and 12.27 ± 0.63 mg RE/g DW, respectively. All MV extracts exhibited potent antioxidant activity against $DPPH^{\bullet}$ (0.106–1.864 mg/mL) and $ABTS^{+\bullet}$ (0.298–17.084 mg/mL). The MV residual aqueous fraction (MVRF) showed significant cytotoxicity against human cancer cell lines, including MCF7 ($IC_{50}$ = 5.47 ± 1.32 µg/mL), HT29 ($IC_{50}$ = 17.48 ± 1.47 µg/mL), and SW480 ($IC_{50}$ = 7.51 ± 0.36 µg/mL). Ultra-performance liquid chromatography-mass spectrometry identified 26 bioactive compounds, with malic acid, caffeic acid, chlorogenic acid, kaempferol-3-glucuronide, and L-tryptophan as the major ones. Molecular docking revealed strong binding affinities of the above compounds to breast (PDB ID: 6CHZ) and colorectal cancer (PDB ID: 1HVY) proteins. Pharmacokinetic and toxicological studies confirmed their safety and efficacy, supporting MVRF as a potential therapeutic agent. These findings highlight MV as a promising candidate for future anticancer research.

**Keywords**: *Marrubium vulgare* L., Antioxidant, Cytotoxicity, UPLC-ESI-QTOF-MS/MS, In silico study

## Supporting Results

## Supporting Information Figures S1-S5

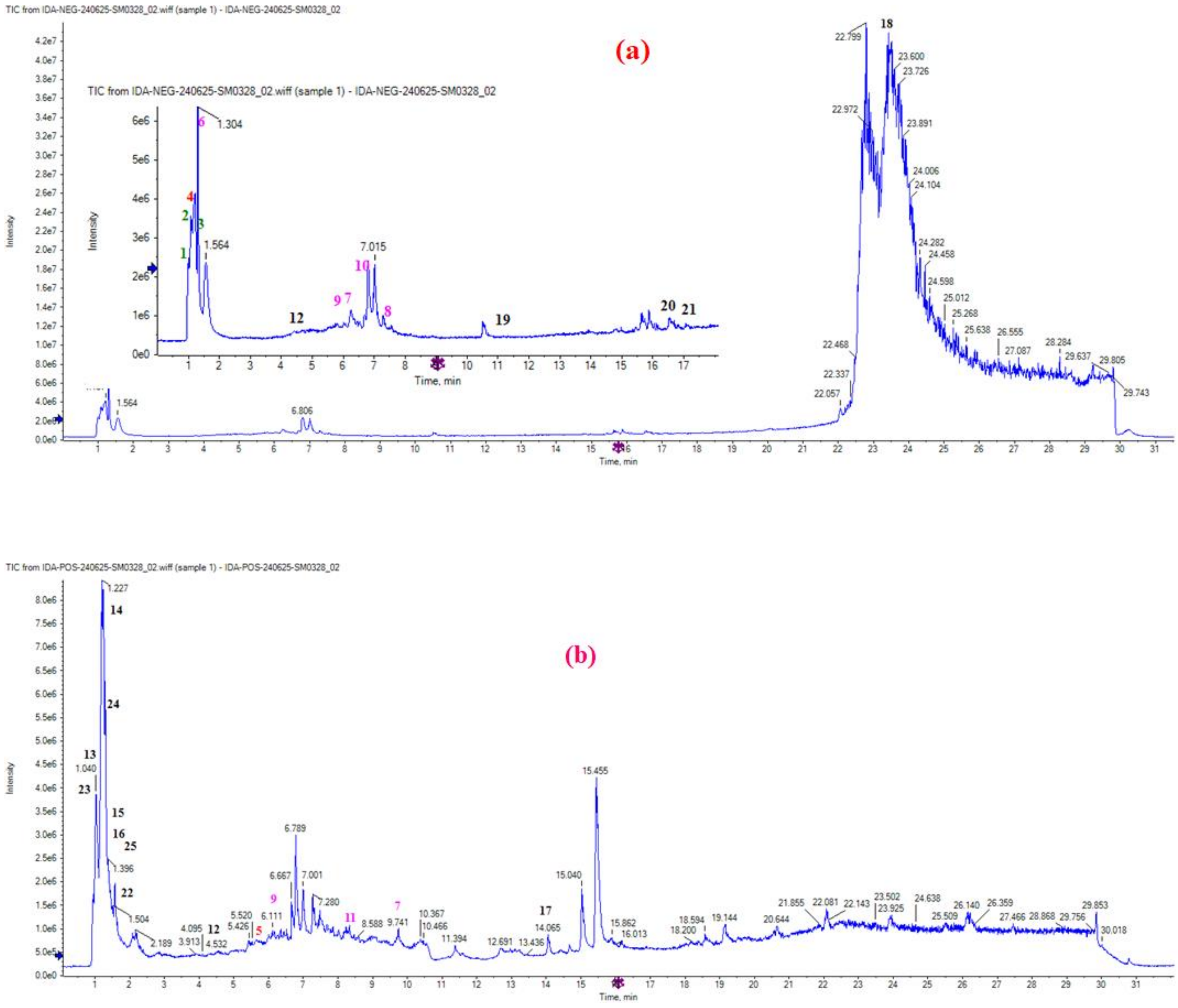


**Fig. S1.** Total ion chromatogram (TIC) of MVRF in (a) ESI$^-$ mode, (b) ESI$^+$ mode

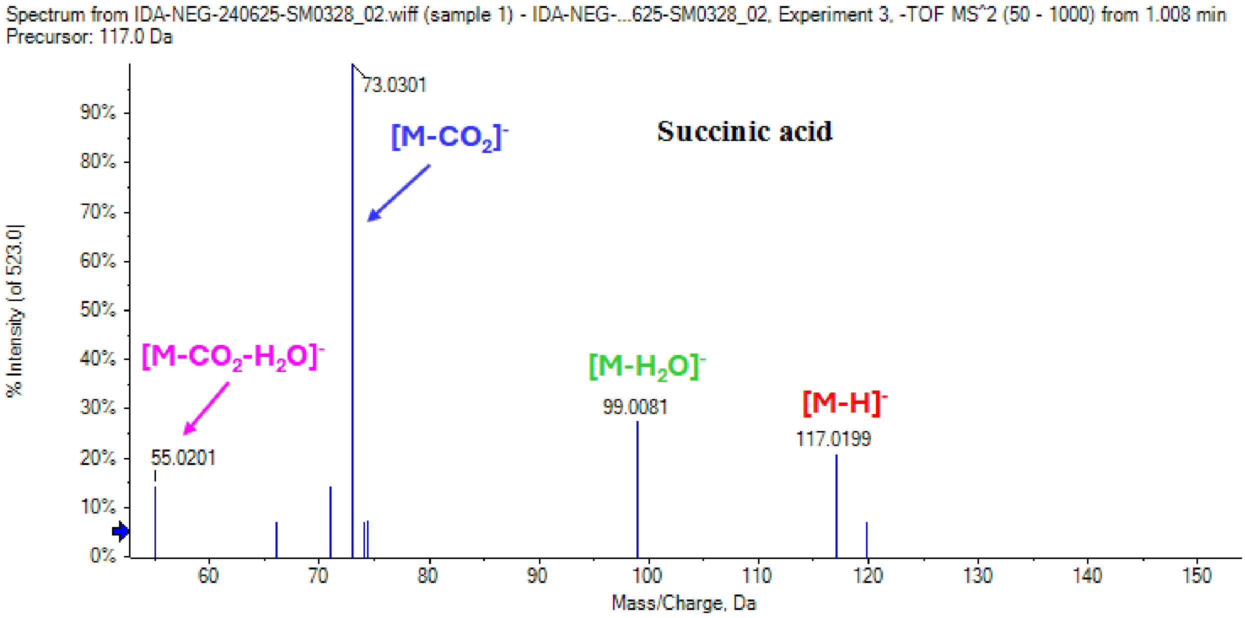
Spectrum from IDA-NEG-240625-SM0328_02.wiff (sample 1) - IDA-NEG-...625-SM0328_02, Experiment 3, -TOF MS^2 (50 - 1000) from 1.008 min
Precursor: 117.0 Da
Succinic acid
73.0301
$[M-CO_2]^-$
$[M-CO_2-H_2O]^-$
55.0201
$[M-H_2O]^-$
99.0081
$[M-H]^-$
117.0199
% Intensity (of 523.0)
Mass/Charge, Da

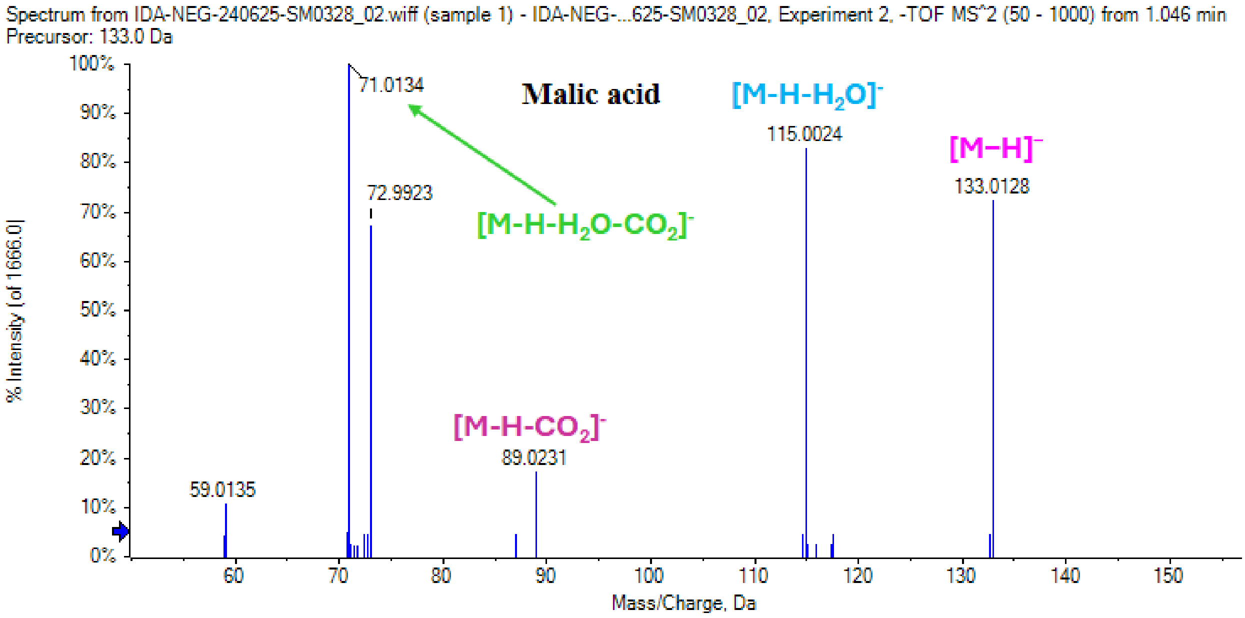
Spectrum from IDA-NEG-240625-SM0328_02.wiff (sample 1) - IDA-NEG-...625-SM0328_02, Experiment 2, -TOF MS^2 (50 - 1000) from 1.046 min
Precursor: 133.0 Da
Malic acid
71.0134
72.9923
$[M-H-H_2O-CO_2]^-$
$[M-H-H_2O]^-$
115.0024
$[M-H]^-$
133.0128
$[M-H-CO_2]^-$
89.0231
59.0135
% Intensity (of 1666.0)
Mass/Charge, Da

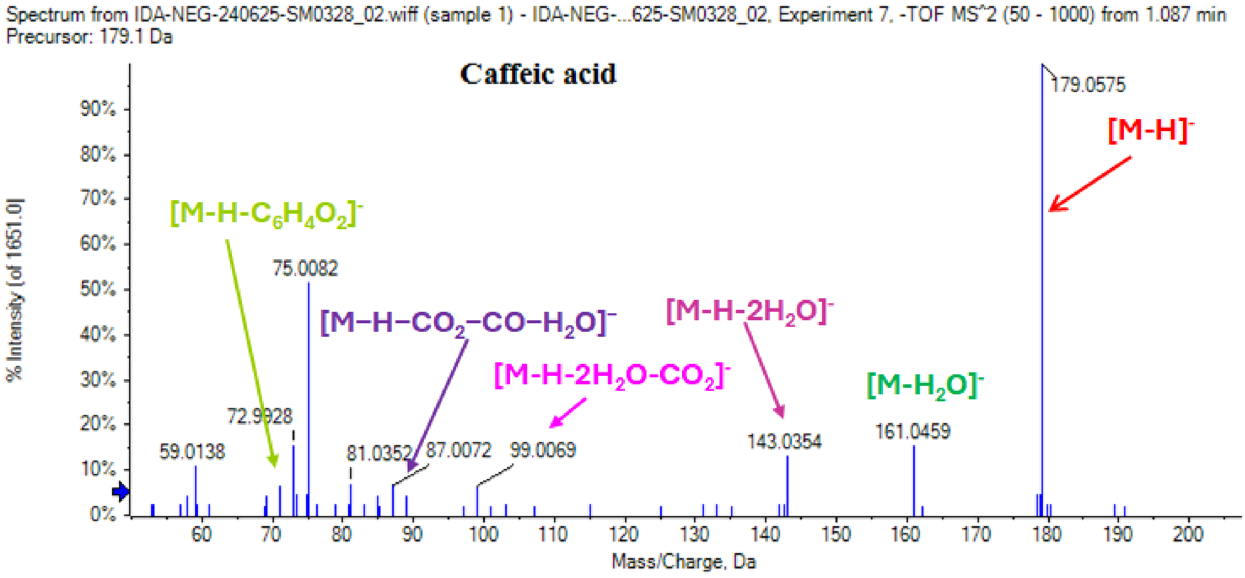
Spectrum from IDA-NEG-240625-SM0328_02.wiff (sample 1) - IDA-NEG-...625-SM0328_02, Experiment 7, -TOF MS^2 (50 - 1000) from 1.087 min
Precursor: 179.1 Da
Caffeic acid
179.0575
$[M-H]^-$
$[M-H-C_6H_4O_2]^-$
75.0082
$[M-H-CO_2-CO-H_2O]^-$
$[M-H-2H_2O]^-$
$[M-H-2H_2O-CO_2]^-$
$[M-H_2O]^-$
72.9928
59.0138
81.0352
87.0072
99.0069
143.0354
161.0459
% Intensity (of 1651.0)
Mass/Charge, Da

Spectrum from IDA-POS-240625-SM0328_02.wiff (sample 1) - IDA-POS-...25-SM0328_02, Experiment 2, +TOF MS^2 (50 - 1000) from 6.087 min
Precursor: 355.1 Da, CE: 35.0

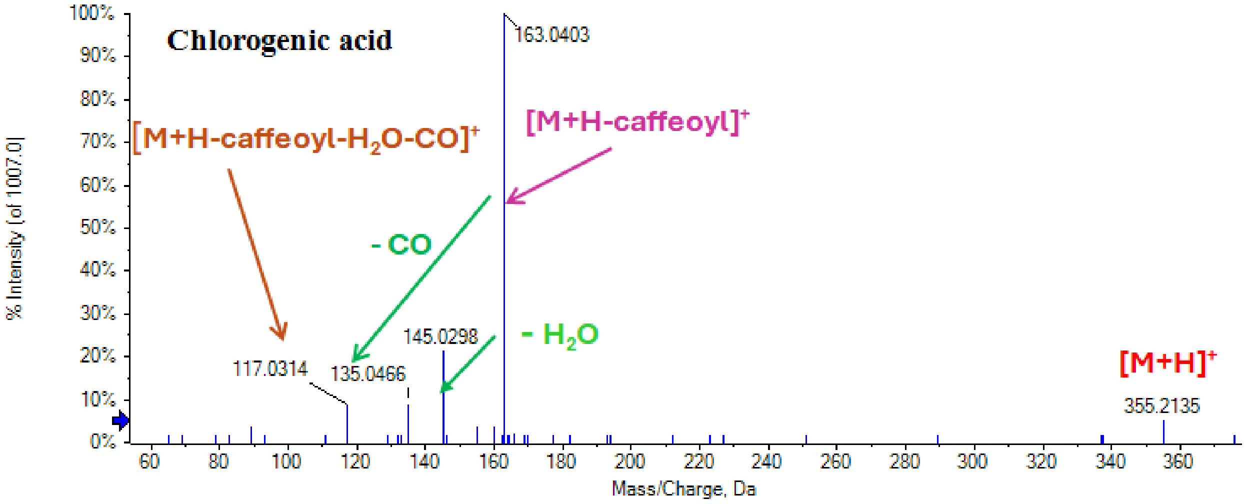


Spectrum from IDA-NEG-240625-SM0328_02.wiff (sample 1) - IDA-NEG-...25-SM0328_02, Experiment 13, -TOF MS^2 (50 - 1000) from 1.309 min
Precursor: 447.1 Da

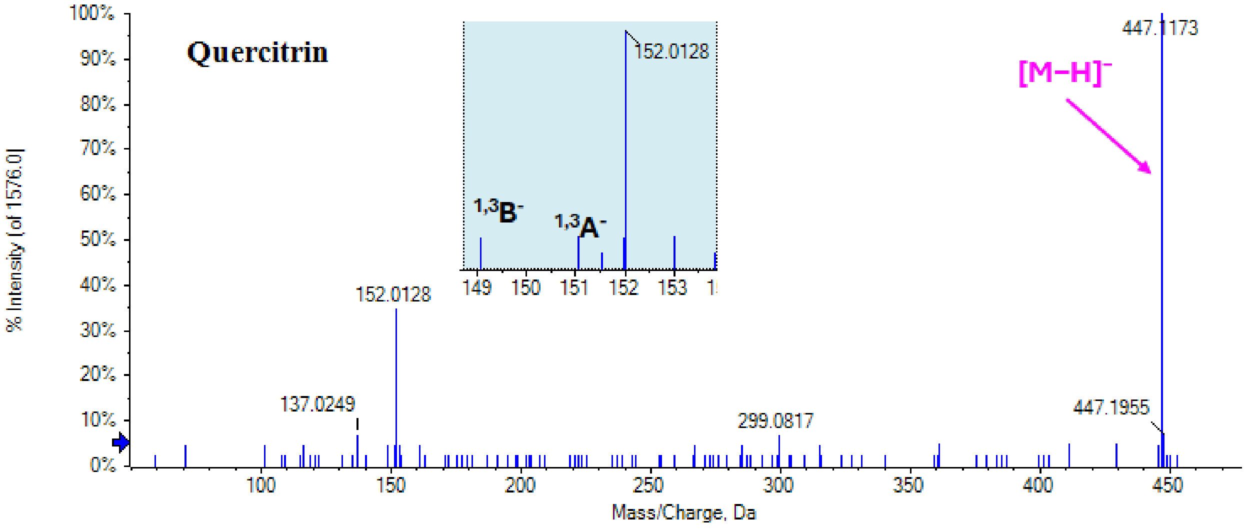


Spectrum from IDA-NEG-240625-SM0328_02.wiff (sample 1) - IDA-NEG-...625-SM0328_02, Experiment 3, -TOF MS^2 (50 - 1000) from 5.785 min
Precursor: 593.2 Da

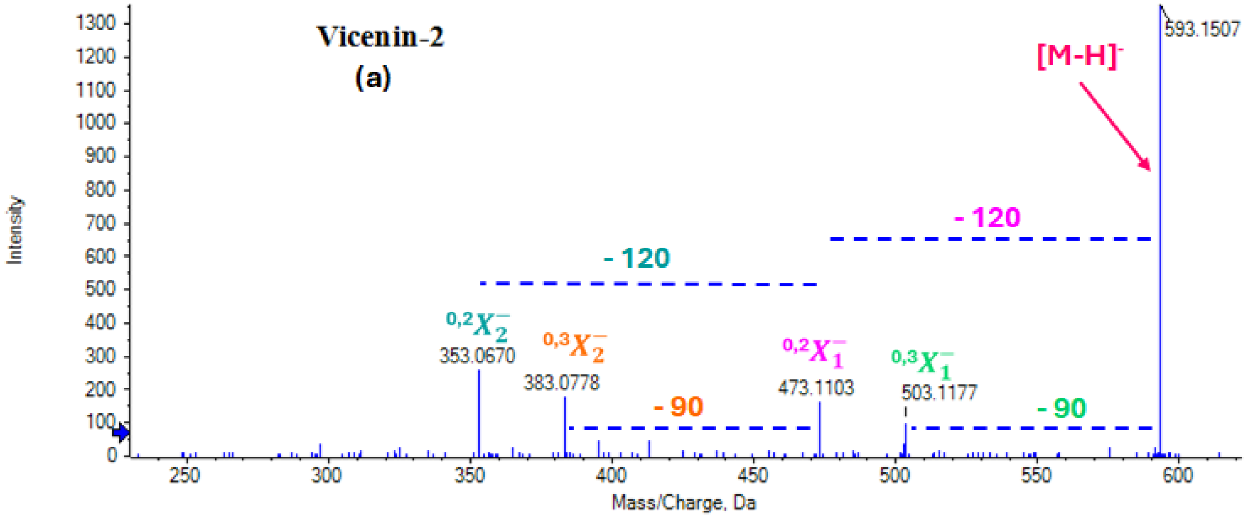

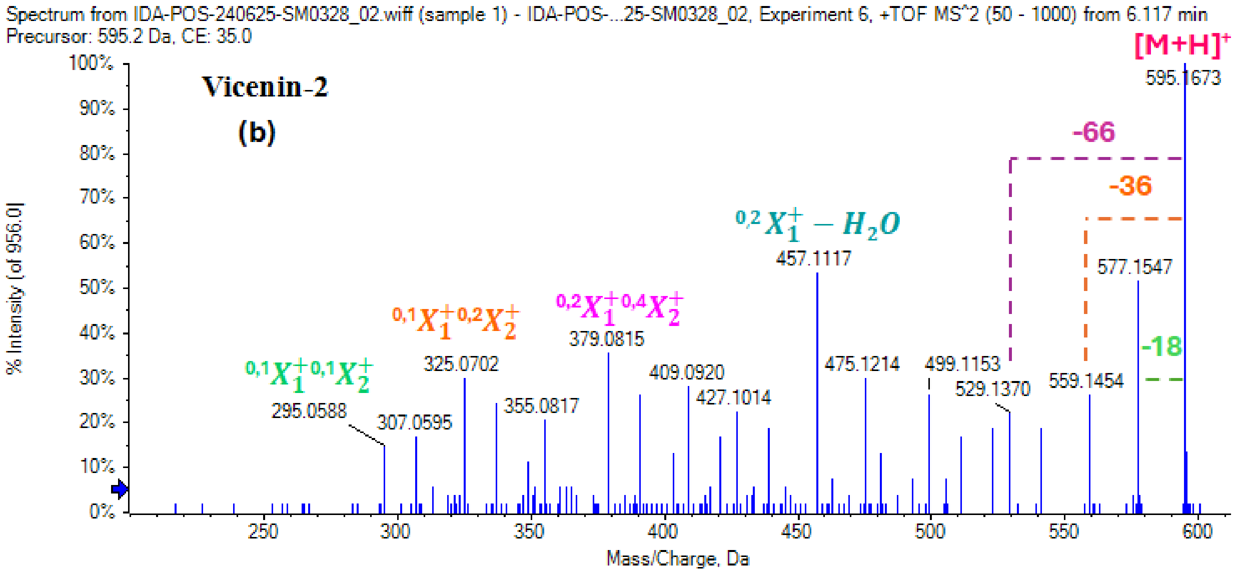
Spectrum from IDA-POS-240625-SM0328_02.wiff (sample 1) - IDA-POS-...25-SM0328_02, Experiment 6, +TOF MS^2 (50 - 1000) from 6.117 min
Precursor: 595.2 Da, CE: 35.0
Vicenin-2
(b)
[M+H]+
595.1673
-66
-36
-18
577.1547
559.1454
529.1370
499.1153
475.1214
457.1117
427.1014
409.0920
379.0815
355.0817
325.0702
307.0595
295.0588
% Intensity (of 956.0)
Mass/Charge, Da

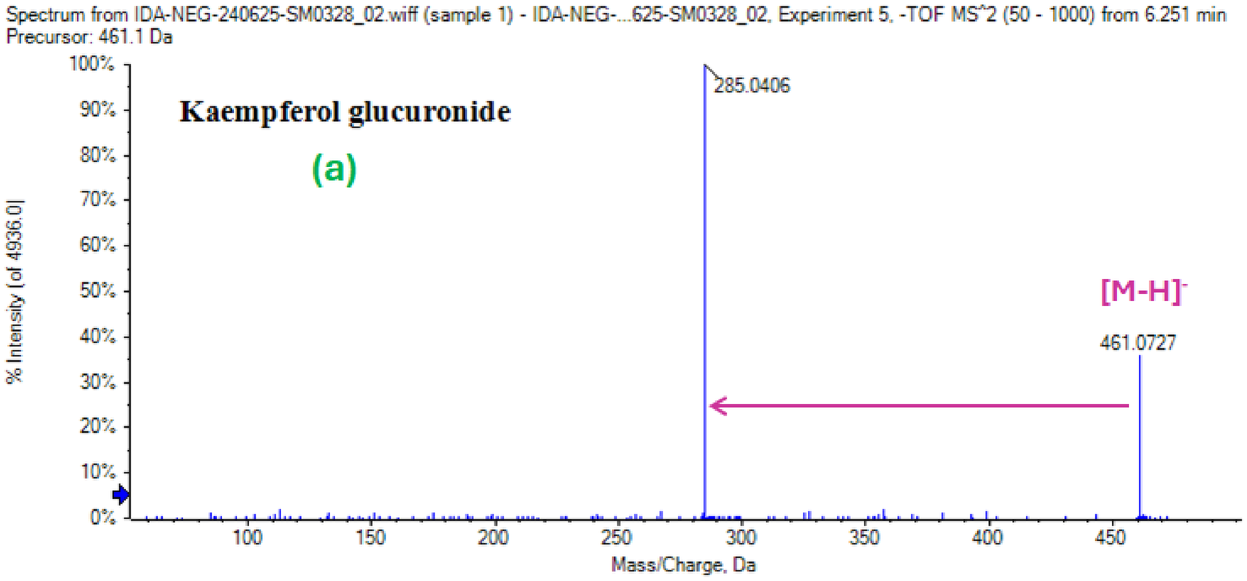
Spectrum from IDA-NEG-240625-SM0328_02.wiff (sample 1) - IDA-NEG-...625-SM0328_02, Experiment 5, -TOF MS^2 (50 - 1000) from 6.251 min
Precursor: 461.1 Da
Kaempferol glucuronide
(a)
285.0406
[M-H]-
461.0727
% Intensity (of 4936.0)
Mass/Charge, Da

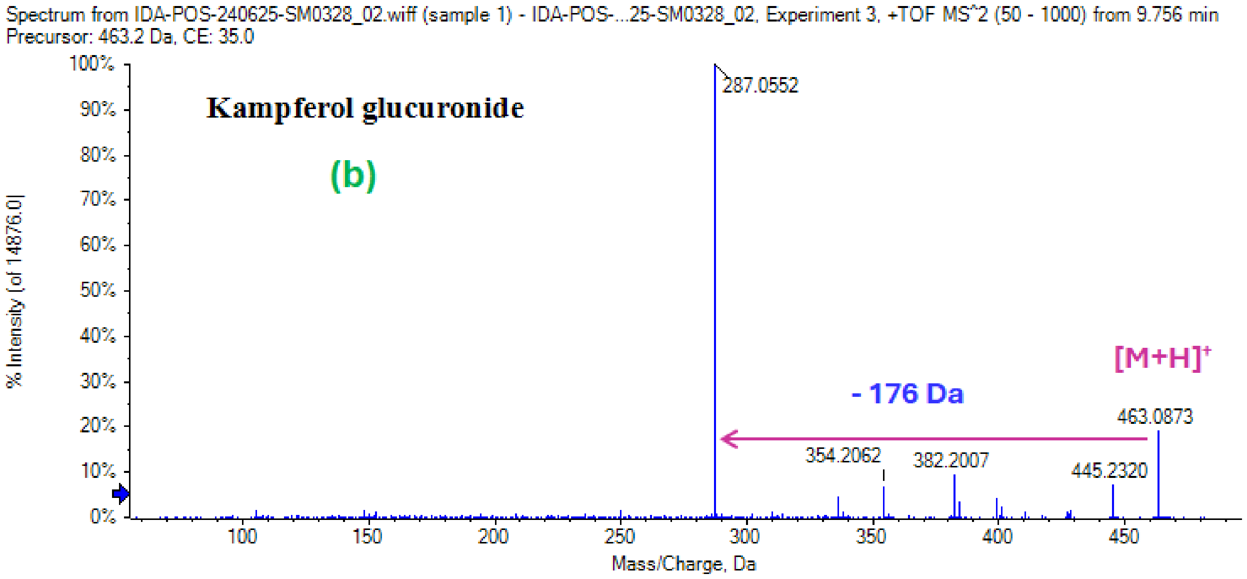
Spectrum from IDA-POS-240625-SM0328_02.wiff (sample 1) - IDA-POS-...25-SM0328_02, Experiment 3, +TOF MS^2 (50 - 1000) from 9.756 min
Precursor: 463.2 Da, CE: 35.0
Kampferol glucuronide
(b)
287.0552
[M+H]+
- 176 Da
463.0873
354.2062
382.2007
445.2320
% Intensity (of 14876.0)
Mass/Charge, Da

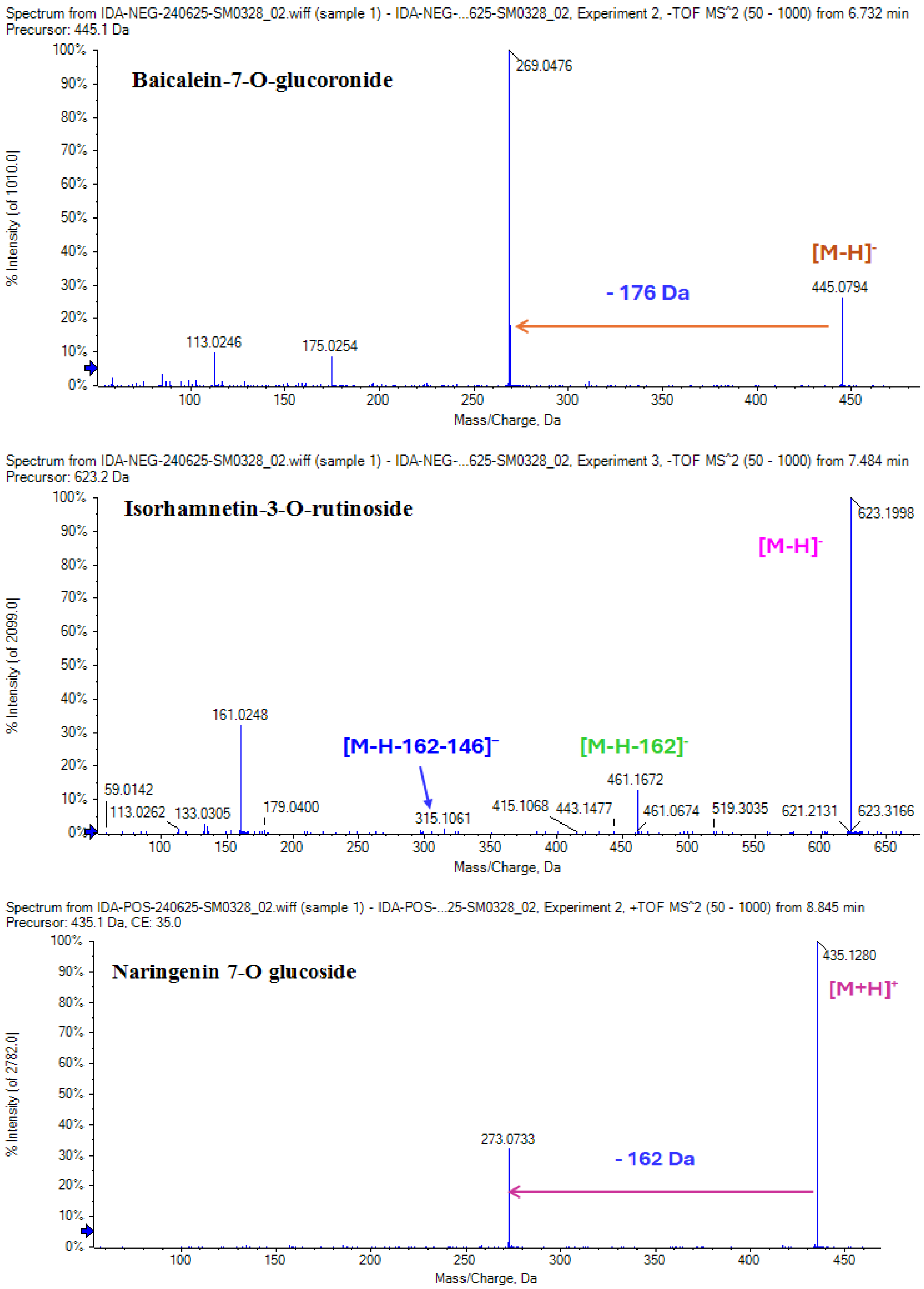


**Fig. S2**. MS/MS spectra and fragmentation patterns of some identified secondary metabolites in MVRF

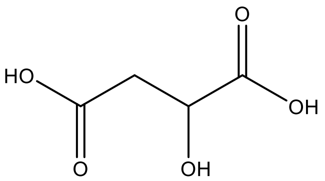

malic acid

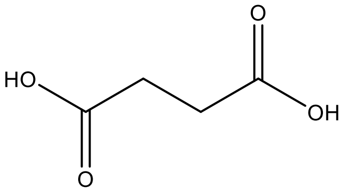

succinic acid

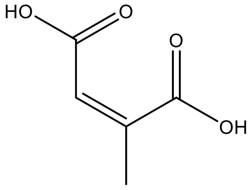

Citraconic acid

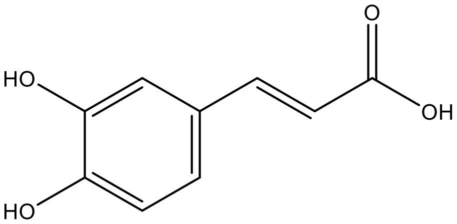

Caffeic acid

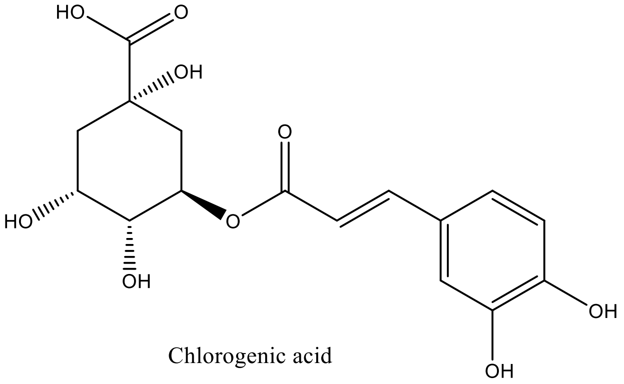

Chlorogenic acid

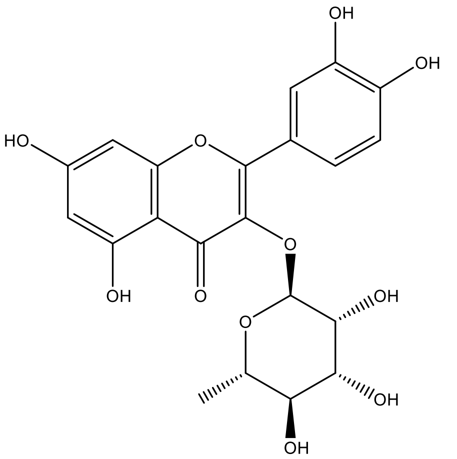

Quercitrin

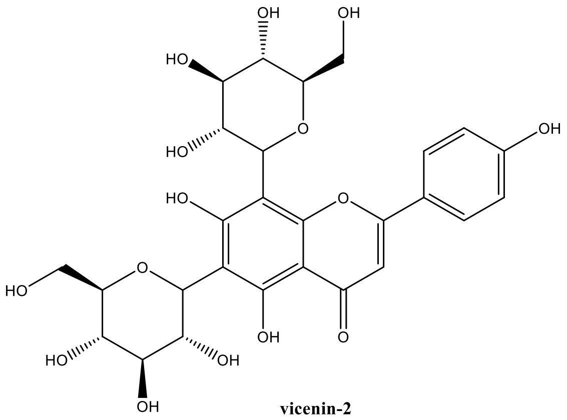

**vicenin-2**

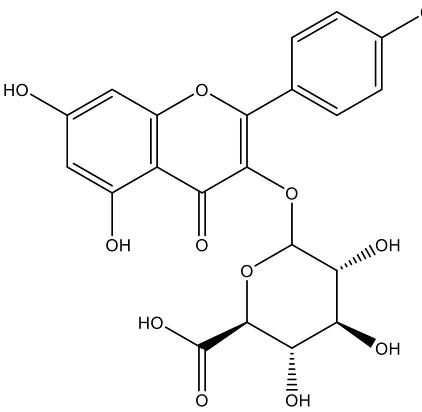

Kaempferol-3-Glucuronide

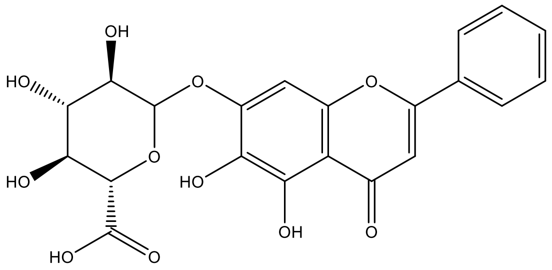

Baicalein-7-O-glucuronide

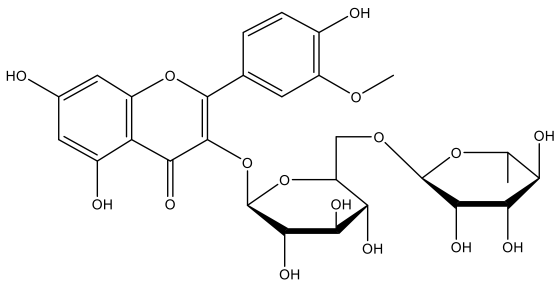

Isorhamnetin-3-O-rutinoside

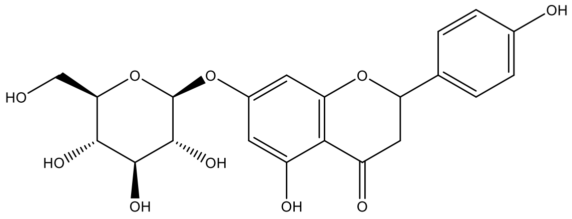

Naringenin 7-O glucoside

**Fig. S3.** Structure of identified phenolic compouds in RFMV by UPLC-ESI-QTOF-MS/MS

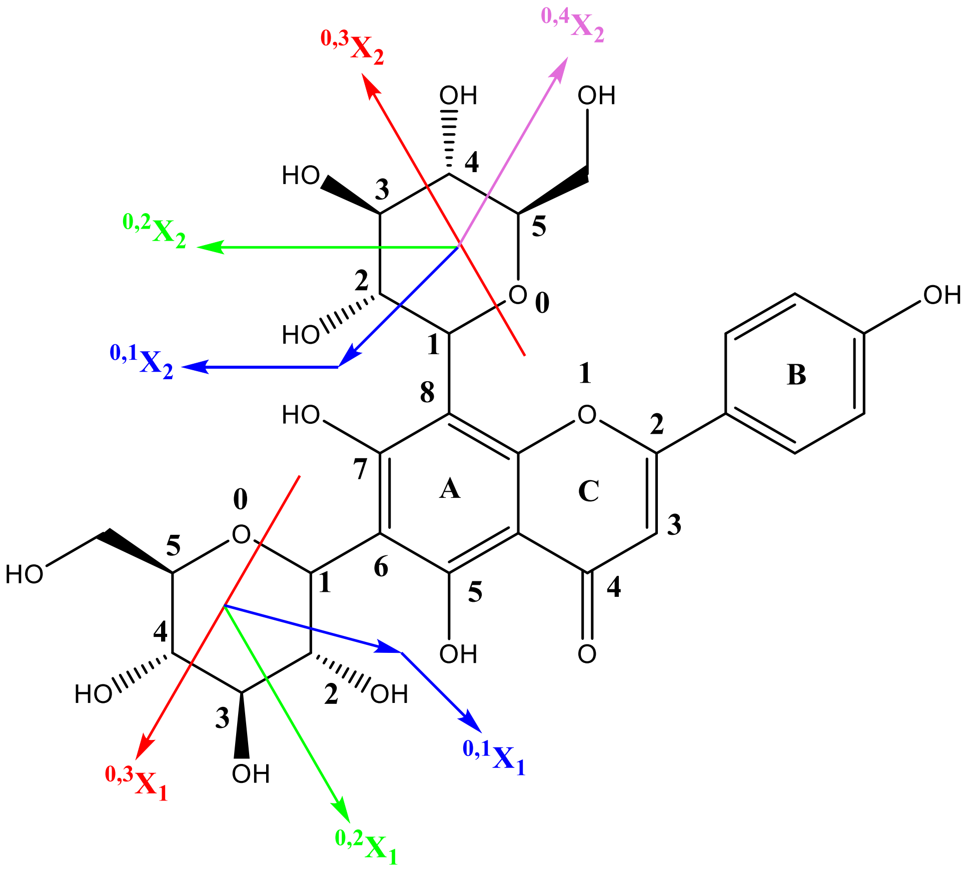


**Fig. S4**. Fragmentation sites of vicenin-2 at C-glycosidic hexose moieties
